# pH Sensitive Hydrogels in Drug Delivery: Brief History, Properties, Swelling, and Release Mechanism, Material Selection and Applications

**DOI:** 10.3390/polym9040137

**Published:** 2017-04-12

**Authors:** Muhammad Rizwan, Rosiyah Yahya, Aziz Hassan, Muhammad Yar, Ahmad Danial Azzahari, Vidhya Selvanathan, Faridah Sonsudin, Cheyma Naceur Abouloula

**Affiliations:** 1 Department of Chemistry, Universiti Malaya, 50603 Kuala Lumpur, Malaysia; rizi_chem1981@hotmail.com (M.R.); ahassan@um.edu.my (A.H.); o_danny@siswa.um.edu.my (A.D.A.); s_vidhya@siswa.um.edu.my (V.S.); 2 Interdisciplinary Research Center in Biomedical Materials, COMSATS Institute of Information Technology, 54000 Lahore, Pakistan; drmyar@ciitlahore.edu.pk; 3 Centre for Foundation Studies in Science, Universiti Malaya, 50603 Kuala Lumpur, Malaysia; sfaridah@um.edu.my; 4 Department of Physics, Faculty of Science Semlalia Marrakesh, Cadi Ayyad University, 40000 Marrakesh, Morocco; cheyma.naceur@ced.uca.ac.ma

**Keywords:** pH sensitive hydrogels, swelling and drug release mechanism, biocompatible materials, drug delivery applications

## Abstract

Improving the safety efficacy ratio of existing drugs is a current challenge to be addressed rather than the development of novel drugs which involve much expense and time. The efficacy of drugs is affected by a number of factors such as their low aqueous solubility, unequal absorption along the gastrointestinal (GI) tract, risk of degradation in the acidic milieu of the stomach, low permeation of the drugs in the upper GI tract, systematic side effects, etc. This review aims to enlighten readers on the role of pH sensitive hydrogels in drug delivery, their mechanism of action, swelling, and drug release as a function of pH change along the GI tract. The basis for the selection of materials, their structural features, physical and chemical properties, the presence of ionic pendant groups, and the influence of their p*K*_a_ and p*K*_b_ values on the ionization, consequent swelling, and targeted drug release are also highlighted.

## 1. Introduction

### 1.1. Brief History of Hydrogels

The term hydrogel dates back to 1894 when this term was used by Lee et al. [1] for colloidal gels of certain inorganic salts. These gels are quite the opposite of the materials which are described by the term hydrogel today [2]. The first ever hydrogel reported in 1949 for biomedical implant was poly(vinyl alcohol) cross-linked with formaldehyde and marketed with the trade name Ivalon [3]. Later in 1958, Danno prepared poly(vinyl alcohol) hydrogels cross-linked by passing gamma irradiation through an aqueous solution [4]. The synthesis of poly(2-hydroxyethyl methacrylate) (pHEMA) gels for contact lens application by Wichterle and Lim [5] in 1960 was the revolution for present day hydrogels. pHEMA exhibited the characteristic properties of modern hydrogels like cross-linked networks that can show swelling upon absorption of excess of water without dissolution and can also retain their shape [6]. The synthesis of pHEMA was also previously reported by du Pont de Nemours scientists in their publication in 1936 describing it as a hard, brittle and glassy polymer [7]. The development of hydrogels for biomedical applications gained particular attention in the 1970s especially in the field of stimuli sensitive hydrogels, the so called smart hydrogels of modern times. The first pH sensitive hydrogel was synthesized in 1971 by Kopecek. The author introduced inorganic groups on the pHEMA backbone to manipulate the pH sensitivity in the pHEMA membranes and studied NaCl permeability control of these membranes as a function of pH [8]. On the basis of history, hydrogels belong to different eras, (i) first generation hydrogels (sixties onwards) are cross-linked hydrogels with relatively high swelling and good mechanical strength, (ii) second generation hydrogels (start of seventies) capable of responding (by swelling or by other events) to specific stimuli such as pH, temperature, biological molecules, etc., (iii) third generation hydrogels comprising of stereo complexed materials (e.g., poly(ethylene glycol)-poly(lactic acid), PEG-PLA cross-linked by cyclodextrin), and present day smart hydrogels with stimuli sensitive tunable properties that can be exploited to make modern day medical devices. In 1980, Lim and Sun [9] explored calcium alginate to prepare microcapsules for successful application in cell encapsulation whereas, later on, Yannas and co-workers [10] explored hydrogels made of collagen and shark fish cartilage for artificial burn dressings. Recently, hydrogels especially gained much attention for organ and tissue reparative and regenerative matrices in the field of tissue engineering [1].

### 1.2. Hydrogels

**Figure 1:**
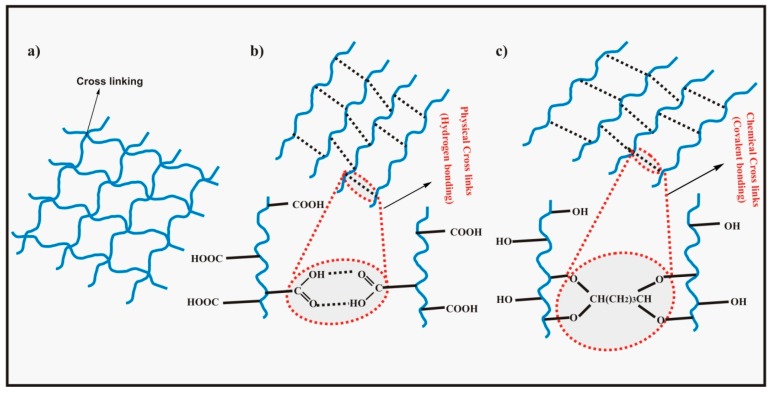
(**a**) Hydrogel matrix; (**b**) physically cross-linked hydrogel matrix; (**c**) chemically cross-linked hydrogel matrix.

Hydrogels are three dimensional cross-linked networks of polymer chains that can absorb and hold lots of water in the interstitial spaces between chains [11]. A hydrogel matrix is shown in Figure 1a. The absorbed solution cannot be removed from swollen hydrogels even under pressure [12]. The absorption of a significant amount of water by hydrogels is attributed to the presence of a large number of hydrophilic groups on the polymer chains such as –NH_2_, –OH, –COOH, –SO_3_H, etc. along with capillary action and osmotic pressure [13] while the hydrogels resist dissolution in the surrounding medium owing to cross-linking between the polymer chains [14]. Also, these hydrophilic groups are responsible for the formation of non-covalent bonds (secondary interactions) of hydrogels with different biological tissues such as mucous membranes and epithelial tissues [15]. Cross-linking in hydrogels may be physical or chemical which prevents hydrogels from dissolution despite absorption of ample amounts of water/physiological fluids [16]. Physical hydrogels involve cross-linking formed by secondary hydrogen bonding between the polar groups on the polymer chains whereas in chemical hydrogels, cross-linking is formed by covalent bonds between different functional groups on the polymer chains facilitated by special cross-linking agents [17]. The difference between physical and chemical cross-linking is shown in Figure 1b,c. Hydrogels are potential materials for biomedical applications such as tissue engineering and drug delivery owing to their softness, hydrophilicity, superabsorbancy, viscoelasticity, biodegradability, biocompatibility, and their similarity with extra cellular matrix. Importantly, hydrogels cause negligible toxicity or tissue damage and do not give inflammatory responses or thrombosis. Another astonishing feature of hydrogels is their reversible responses to different stimuli like pH, temperature, electric field, magnetic field, ionic strength of solution, and biological molecules [18,19] that make them particularly important for a wide range of biomedical applications [20].

#### 1.2.1. Unique Properties of Hydrogels

Hydrogels, when fully swollen, show some unique properties such as being soft and rubbery and having low interfacial tension with water and biological fluids. These properties, especially for the natural hydrogels, make them similar to extra cellular matrix in living tissues [13,21]. For hydrogels, there are less chances of negative immune response due to low interfacial tension with body fluids which reduces cell adhesion. Many hydrogels have enhanced tissue permeability and drug residence time owing to their mucoadhesive and bioadhesive properties which make them very good vehicles for drug delivery [16,22].

The beauty of natural polymers is that they are non-toxic, biodegradable, biocompatible, abundant, and cheap. However, natural polymer based hydrogels are poor in mechanical strength. On the other hand, biocompatible synthetic polymer based hydrogels have good mechanical strength but are expensive, non-biodegradable, and susceptible to shear degradation. To obtain hydrogels with better properties (additive of the two types of polymers), natural polymers (e.g., polysaccharides) have been blended with biocompatible synthetic polymers (e.g., acrylic polymers). This helps to facilitate high drug concentration in the targeted region/tissue and controlled drug release for an extended period of time. Alternatively, a grafting reaction may be utilized to bind synthetic monomers/polymer onto natural polymer chains to get improved properties [12]. The pores and their sizes in the hydrogel structure play a very important role in deciding their ability to load and release the drug in physiological fluids in vitro and/or in vivo. Porosity, a primary characteristic of hydrogels, can be controlled by tailoring their affinity with water and cross-linking. The affinity of hydrogels for water in turn varies with the number of hydrophilic groups along the polymer chains whereas the cross-linking density is dictated by the concentration of the cross-linker and the time for cross-linking. The release of loaded drug may take place by several mechanisms such as swelling control, diffusion control, environmental sensitivity control, and chemically controlled release [23].

#### 1.2.2. Classification of Hydrogels

**Figure 2:**
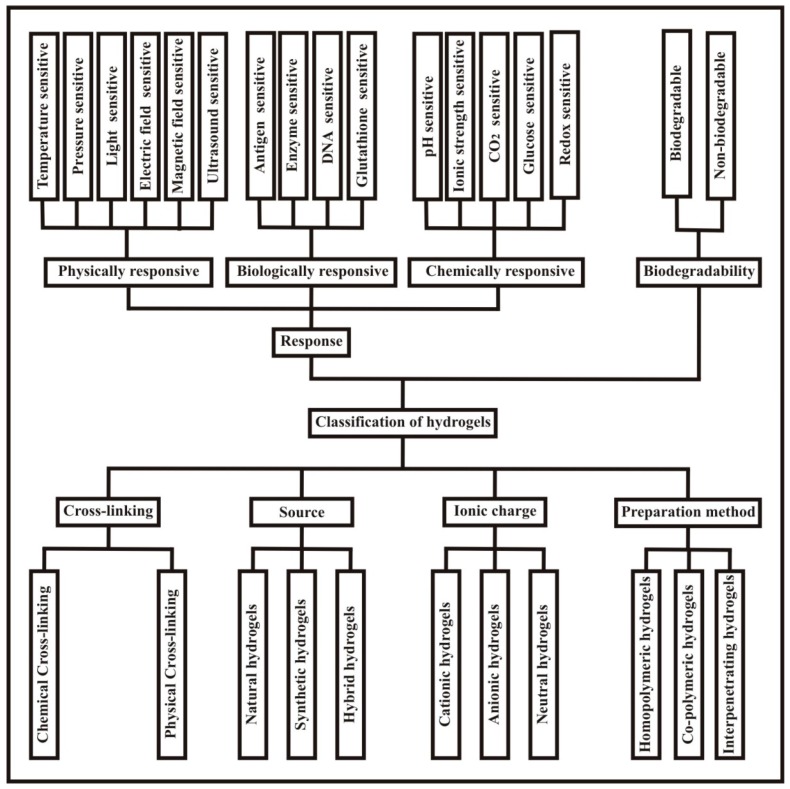
Complete classification of hydrogels based on different factors.

Hydrogels are classified based on various factors like biodegradability, type of cross-linking, source, ionic charge, preparation method, physical properties, and the response nature of the hydrogels to external stimuli [13]. The complete classification of hydrogels is shown in Figure 2.

#### 1.2.3. Stimuli Sensitive/Responsive Hydrogels

**Figure 3:**
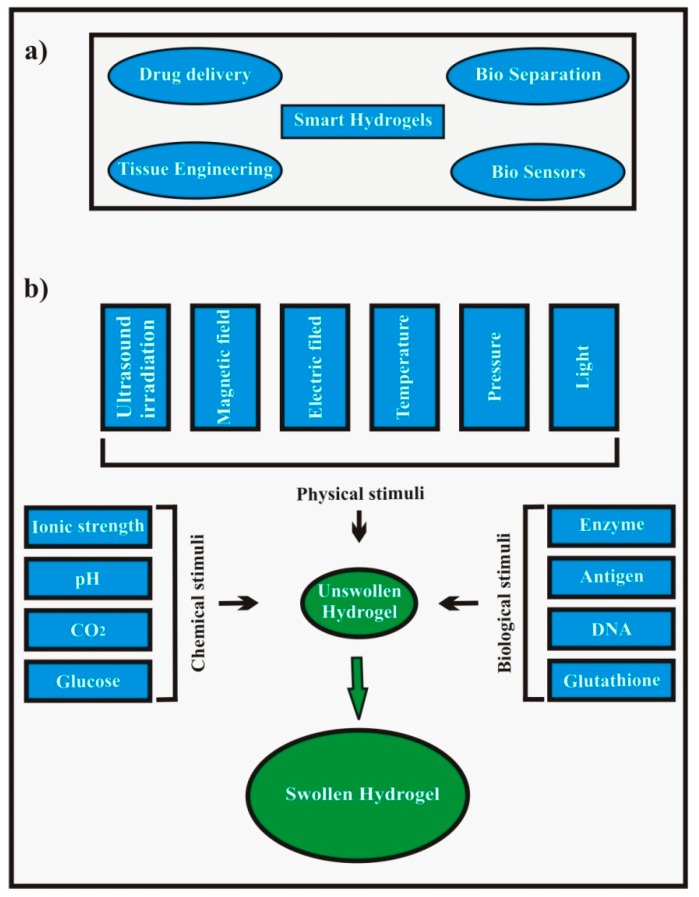
(**a**) The four broad areas of smart hydrogels, (**b**) stimuli sensitive swelling of hydrogels along with their categories, (i) physical stimuli, (ii) chemical stimuli, and (iii) biological stimuli.

Hydrogels which are sensitive to specific environmental changes and show responses by changing their shape or volume when exposed to certain conditions are regarded as stimuli sensitive hydrogels. These are sensitive to (i) physical stimuli such as light, pressure, temperature, electric field, magnetic field, ultrasound, (ii) chemical stimuli such as pH, redox, ionic strength, CO_2_, glucose, and (iii) biological stimuli such as enzymes, [24] antigens, glutathione, and DNA. The main applications of smart hydrogels are summarized in Figure 3a and the trigger factors for the swelling of stimuli sensitive hydrogels are categorically shown in Figure 3b.

These stimuli can also be categorized as internal or external stimuli based on their source at the time of application to the hydrogels in vivo. Chemical and biological stimuli belong to the former category whereas physical stimuli belong to the latter category except temperature which may be an external or internal stimulus [25]. The term ‘smart’ or ‘intelligent’ has been coined for these hydrogels in the sense that they perceive the stimulus and respond by a change in their physical and/or chemical behavior leading to release of the entrapped drug [26]. Examples of different stimuli sensitive hydrogels and their mechanisms of response are laid out in Table 1.

Amongst the stimuli responsive hydrogels, pH sensitive hydrogels are the most studied hydrogels. Collapse and swelling are the abrupt changes that occur when stimuli sensitive hydrogels are exposed to a certain stimulus leading to volume phase transition. The rate at which hydrogels respond depends upon their size, shape, cross-linking density, number of ionic groups, and composition which can be tailored by varying these factors. The response rate increases with increasing pore size and number of ionic groups and by decreasing their size and cross-linking density [44]. Another interesting class of smart materials is shape memory polymeric hydrogels that have a permanent shape of which there is a code to restore the original shape in the presence of a specific stimulus. Shape memory polymeric hydrogels require two basic elements, (i) the polymer should have a property of stimuli controlled phase transition, and (ii) the polymer should have a memory code (physical or chemical) that can help to recover the original shape in the presence of a specific stimulus. Guo et al. prepared pH controlled shape memory hydrogels comprising two cross-linked polymers of acrylamide/deoxyribonucleic acid (DNA) that can function co-operatively. At pH 5, the specific permanent shape of the hydrogel is stabilized by the cross-linked poly(acrylamide) and duplex bridges in DNA. At pH 8, the hydrogel deforms to give a quasi-liquid state whereas the DNA duplex bridges serve as a memory code, a feature that helps to re-assemble it into the original permanent shape when exposed again to pH 5 [45].

Reduction sensitive hydrogels are a special class of smart/intelligent hydrogels that respond to reduction conditions due to higher levels of glutathione tripeptide (GSH) and are characterized by the presence of disulfide linkages located on the main chain/side chain or as a cross-linker. At low concentration of GSH (2.0–20 μM) in the body circulation and extracellular matrix (ECM), disulfide bonds are stable while these bonds undergo quick cleavage (owing to a thiodisulfide exchange reaction) under a GSH concentration as high as 0.5–10 mM in the intracellular milieus. The GSH concentration is even four times more in the tumor tissue than the normal tissue. This cleavage of disulfide linkages is responsible for the release of drug or bioactive agents in the intracellular matrix [46]. Yu et al. fabricated reduction sensitive hydrogels by including [poly(ethylene glycol) monomethyl ether]-graft-[disulfide linked poly(amido-amine)] (mPEG-*g*-SS-PAA) with α-cyclodextrin. They loaded the bovine serum albumin (BSA) and studied its reduction sensitive release profile in the intracellular matrix [41].

#### 1.2.4. Properties of pH Sensitive Hydrogels

pH sensitive swelling is attributed to ionic hydrogels having charge carrying pendant groups and is controlled by many factors such as ionic charge, p*K*_a_ or p*K*_b_ values of ionizable groups, degree of ionization, hydrophilicity, polymer concentration, and pH of the swelling medium. Among these factors, pH and the nature of pendant groups are the key factors for controlling the properties of pH sensitive hydrogels. Cationic hydrogels like chitosan and poly(ethylene imine) [35], swell at low pH (acidic medium) due to protonation of amino/imine groups. The protonated positively charged moieties on the polymer chains cause repulsion and hence are responsible for swelling. These types of hydrogels can be used for drug (antibiotic) delivery to the stomach during ulceritis or as carriers for an injectable drug delivery system. Anionic hydrogels like carboxymethyl chitosan swell at higher pH (basic medium) due to ionization of the acidic groups. As a result, the ionized negatively charged pendant groups on the polymer chains cause repulsion leading to swelling. This property of hydrogels can be exploited for drug delivery at pH 7.4 in the intestine [47]. Another novel approach which proves to be successful for drug delivery applications is use of the polyelectrolyte complex (PEC) hydrogels which dismisses the usage of toxic covalent cross linkers. PEC hydrogels are mainly comprised of two components, (i) a cationic polymer like chitosan, and (ii) an anionic polymer like carboxymethyl chitosan and are stabilized by electrostatic interaction between these opposite charges in the blend. Zaino et al. prepared pH sensitive PEC hydrogels comprising of *N*-trimethyl chitosan (cationic component) and *N*-carboxymethyl chitosan (anionic component) to study the drug delivery profile of dexamethasone [48].

#### 1.2.5. Theory and Swelling Mechanism of pH Sensitive Hydrogels

Swelling of hydrogels upon exposure to water/physiological fluids depends upon the osmotic pressure within the hydrogels caused by the hydrophilicity of the constituting polymers, the static charges on the polymer, and the counter ions within the hydrogel matrix. Swelling of hydrogels involves three steps: First the diffusion of water into the hydrogel network, second the loosening up of the polymer chains upon hydration and third the expansion of the hydrogel network when the polymer chains relax [49,50]. The attraction towards water molecules of the hydrophilic and polar groups leads to absorption and this is called primary bound water. As a result, the hydrogels swell and the exposed hydrophobic moieties interact with water molecules which is called secondary bound water. Additional water will move into the hydrogel under the influence of an osmotic driving force opposed by the cross-links owing to an elastic retractive force. This additionally imbibed water is called free water and the hydrogels reach their equilibrium swelling [1]. At this equilibrium, there is a balance between the elastic retractive forces of the chains and osmotic pressure. This is explained by the Flory and Rehner theory. According to this theory, swelling is a function of the elastic nature of polymer chains and the thermodynamic based compatibility of water molecules with the polymer chains. Volume phase transition occurs when stimuli responsive hydrogels are exposed to a certain stimulus [44]. The swelling of ionic hydrogels is controlled mainly by two factors, (i) properties of the polymer constituting the hydrogel such as cross-linking density, hydrophilicity, hydrophobicity, concentration, ionic charge, p*K*_a_ value of the acidic pendant group or p*K*_b_ value of the basic pendant group, and (ii) properties of the swelling medium such as ionic strength, pH and counter ion [13].

**Figure 4:**
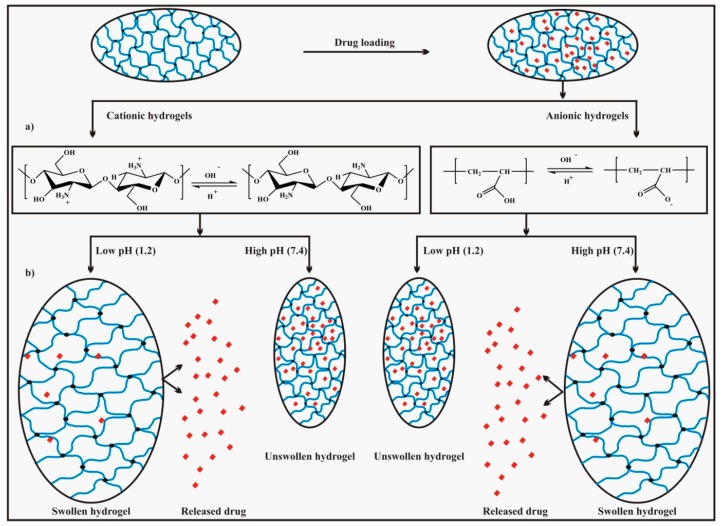
(**a**) pH dependent ionization of specific acidic or basic functional groups on hydrogel chains responsible for swelling, (**b**) pH dependent swelling and drug release mechanism.

The swelling of hydrogels having acidic or basic pendant groups on the polymer chains depends upon the pH of the surrounding medium relative to the respective p*K*_a_ and p*K*_b_ values of the pendant groups. In the case of the anionic network (e.g., having carboxylic, –COOH pendant groups), when the pH value of the surrounding medium is greater than the p*K*_a_ value of the acidic pendant groups on the polymer chains, ionization of the acidic group takes place leading to the production of fixed negative charges on the polymer chains and mobile positive charges in the solution. As a result, there is an increase in (i) the hydrophilic nature of the hydrogels, (ii) the number of fixed negative charges, and (iii) the electrostatic repulsion between the chains leading to swelling of the hydrogel network and vice versa (when the pH is less than the p*K*_a_). On the other hand, for the cationic network (e.g., having amino, –NH_2_ pendant group), if the pH of the surrounding medium is less than the p*K*_b_ value of the pendant basic groups, protonation (ionization) of the pendant group takes place which results in an increased number of fixed positive charges on the polymer chains and mobile negative charges in the solution. Consequently, this causes swelling due to an increase in (i) the hydrophilic nature of the polymer chains, (ii) the number of fixed positive charges, and (iii) the electrostatic repulsion between the chains and vice versa (the pH is greater than the p*K*_b_) [26,44]. The pH dependent ionization of specific acidic or basic functional groups on hydrogels chains responsible for swelling is shown in Figure 4a.

Stimuli responsive hydrogels that show reversible temperature dependent swelling behavior are termed thermoresponsive hydrogels. Based on their response to temperature, thermoresponsive hydrogels are classified as (i) positive thermoresponsive hydrogels like poly(acrylamide) which swell above their characteristic upper critical solution temperature (UCST) and vice versa, (ii) negative thermoresponsive hydrogels like poly(*N*-methacrylamide) which swell below their characteristic lower critical solution temperature (LCST) and vice versa [25,44], and (iii) thermo-reversible hydrogels like (poly(ethylene oxide)-poly(propylene oxide)-poly(ethylene oxide)) (PEO-PPO-PEO) which undergo sol-gel phase transition below and above the critical solution temperature (CST) instead of the swelling-shrinking transition [13]. The UCST response in poly(acrylic acid) and poly(acrylamide) is primarily due to the presence of secondary hydrogen bonding which dominates at low temperature and keeps the hydrogel matrix shrunk whereas, with increasing temperature, hydrogen bonds weaken and the hydrophilic ends become exposed leading to the phenomenon of swelling [51,52]. The hydrophobic interactions between polymer chains and water are responsible for the LCST response of poly(*N*-methacrylamide). The polymer network becomes more ordered and shrinks/collapses with the rise in temperature whereas water molecules become less ordered and are removed from the polymer network [52,53]. Specific van der Waals forces like hydrogen bonding and hydrophobic interactions are responsible for the thermo-reversible behavior of hydrogels at CST. In solution (sol form), there exists hydrogen bonding between the polar groups of the polymer chains whereas this bonding becomes unfavorable at CST and polymer–polymer, water–water interactions dominate, excluding the water from the hydrogel by dehydration. This leads to an overall increase in the entropy of the system and consequently shrinkage of the hydrogel (gel form) [13].

#### 1.2.6. Drug Release Mechanism of pH Sensitive Hydrogels

There are different release mechanisms of entrapped/encapsulated drug in hydrogels such as diffusion controlled, swelling controlled, and chemically controlled mechanisms. The diffusion controlled mechanism is the most acceptable one and its drug release model follows Fick’s law of diffusion. Porosity of the hydrogels is related to the diffusion coefficient of the hydrogels if the molecular dimensions of the drug molecules are much smaller than the pore size of the porous hydrogels. When the pore size in the hydrogels and the size of the drug molecules are comparable, the release of the drug molecules is hindered by the cross-linked polymer chains. As a result, the diffusion coefficient is decreased. If the rate of drug release exceeds the rate of swelling then drug release follows a swelling controlled mechanism [54]. This involves absorption of water molecules followed by desorption of the drug. The resistance of dry (glassy) polymer hydrogels to undergo a change in shape and increase in volume during the hydration process controls the rate of drug release which in turn can be controlled by the composition of the hydrogels and the cross-linking density. Free interstices between intermolecular chains allow the solvent to penetrate the surface of the hydrogels when they are in contact with water or certain physiological solutions. The solvent moving in develops a stress responsible for the increase in distance between the polymer chains (polymer chain relaxation) leading to swelling. This swelling process is accompanied by desorption of the drug and its controlled release [26,55].

If the entrapped molecules in the hydrogels network are smaller such as peptides/proteins, their diffusion is easy and their release takes place by a diffusion controlled mechanism whereas for larger entrapped molecules like plasmid DNA, diffusion is not easy and their release from the matrix follows a chemically controlled mechanism [56]. Drug release due to the reactions of hydrogels (hydrolytic or enzymatic degradation of polymer chains) is said to follow a chemically controlled mechanism. It is further categorized as (i) a kinetically controlled release mechanism, and (ii) a reaction diffusion controlled mechanism. In the former case, there is negligible diffusion and the bond cleavage in the polymer chains (polymer degradation) dominates which is the rate determining step, whereas for the latter case diffusion as well as polymer reactions (polymer degradation) collectively explain the drug release [54]. The general mechanism of pH dependent swelling as well as drug release is shown in Figure 4b.

## 2. Base Materials for pH Sensitive Hydrogels

### 2.1. Natural Hydrogels

Hydrogels derived from natural polymers of plant or animal origin and those obtained from bacteria, yeast, and fungi are termed natural hydrogels [57]. Features of natural polymer based hydrogels such as non-toxicity, low cost, abundance, biocompatibility and biodegradability, and similarity to extra cellular matrix have attracted researchers in pursuing them for their use in different biomedical applications [12]. However, these advantages are offset due to the fact that the properties and microstructure of the natural polymers are difficult to control experimentally in a reproducible manner: (i) firstly, their poor mechanical properties (ii) secondly, the variation in composition from batch to batch. This is the reason why chemical modification and/or blending with other synthetic polymers is sometimes required to tailor improved properties such as solubility, mechanical properties, sensitivity to specific stimulus, and muco-adhesivity [58].

#### 2.1.1. Chitosan

**Figure 5:**
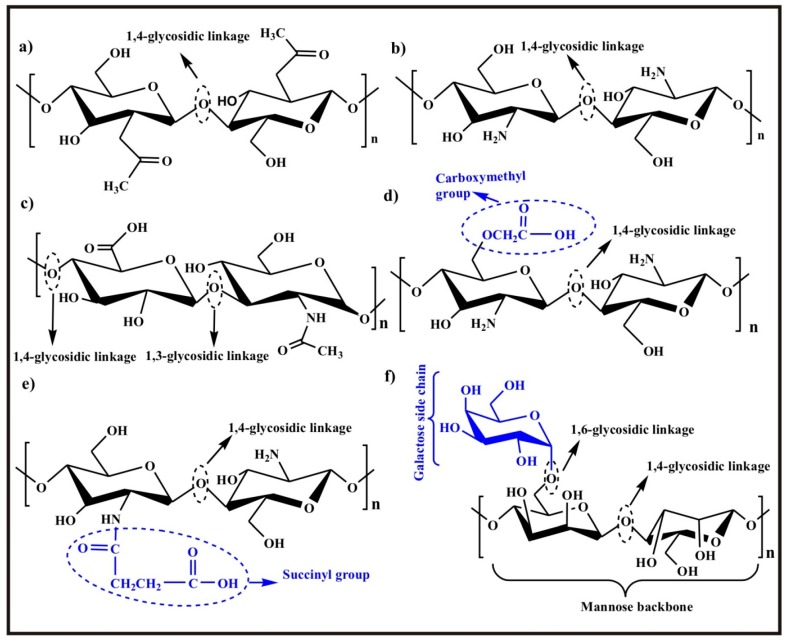
Structure of (**a**) chitin, (**b**) chitosan, (**c**) glycosaminoglycan, (**d**) carboxymethyl chitosan, (**e**) *N*-succinyl chitosan and (**f**) guar gum.

The history of chitosan, CS has its origin as old as 1859 when Rouget mentioned the deacetylated form of chitin as CS [59]. CS consists of *N*-acetylglucosamine and d-glucosamine repeating units connected through 1,4-glycosidic linkages. The proportion of the former unit depends upon the degree of deacetylation. It is a cationic polymer which is obtained by partial deacetylation of chitin and is biocompatible, biodegradable, and non-toxic [20,60]. Chitin is found in the exoskeleton of crustaceans such as shrimps, crabs, lobsters, etc. and is the second most abundant natural polymer after cellulose [61,62]. The chemical structure of chitin is shown in Figure 5a. Yeast and fungi are also sources of chitin and the cell walls of some fungi such as Mucor, Absidia, and Rhizopus are also direct sources of CS [63]. The chemical structure of CS is shown in Figure 5b. CS and its derivatives have attracted researchers to pursue their applications in different fields: biomedical, cosmetics, textile, biotechnology, drug delivery, etc. owing to their remarkable properties of being antifungal, antibacterial [64], hemostatic, healing acceleration [2], and antioxidant. The cationic CS chains interact with negatively charged bacteria responsible for the disruption of the cell wall leading to antimicrobial properties of CS [65]. The mechanism involved in antimicrobial activity proceeds via a given sequence (i) cationic chains of CS bind with sialic acid in phospholipids preventing the transport of microbiological compounds, and (ii) oligomeric CS enters the cells of micro-organisms which converts DNA into RNA leading to ceasing of growth [66].

CS has a structure proxy to glycosaminoglycan which is a major component of extra cellular matrix. Glycosaminoglycan facilitates cell proliferation and attachment and enhances the biocompatibility of cell/tissue and biomaterials [67]. The chemical structure of glycosaminoglycan is shown in Figure 5c. CS is only soluble in acidic aqueous solution because the amino group on the CS chains gets protonated in this medium and hence is responsible for its solubility. The solubility of CS is controlled by many factors including (i) degree of deacetylation, (ii) distribution of the acetyl group on the CS chains, (iii) the conditions of isolation, (iv) pH, (v) extent of ionization, and (vi) nature of acid. However, CS with a degree of deacetylation more than 50% starts dissolving in acidic medium. A very interesting fact about CS is that it can also be dissolved at neutral pH in the presence of glycerol-2-phosphate [68]. Poor solubility and mechanical strength of CS restrict its wide range biomedical applications. Due to these drawbacks, it undergoes deformation under external pressure leading to extensive swelling in acidic aqueous solutions [62,67].

CS is important because of some of its biological activities such as cholesterol lowering activity, antihypertension activity, activation of immune response, phytoalexin elicitor activity, as well as its therapeutic properties like inhibition of micro-organism growth, promotion of hemostasis, epidermal cell growth, and pain alleviation [61]. Additionally, CS shows good anti-ulcer and antimicrobial properties. CS bears a good muco-adhesive property owing to its cationic nature enhancing the residence time in the intestine as well as the bioavailability of drugs in the GI tract. The positive charge of CS interacts electrostatically with the negatively charged sialic acid of mucin which helps its adhesion with the mucous layer [47]. To facilitate paracellular transport of drugs, CS has the ability to open the tight junction between intestinal epithelial cells [69] and promote intestinal adsorption [47]. This is initiated by electrostatic interaction between CS and integrin receptors on the cell membranes [70]. The –NH_2_ group on the CS chains is protonated only at acidic pH and deprotonated at neutral pH because the p*K*_a_ value of the amino group is nearly 6.5. The pH value of the duodenum is in the range 6.5–7.0 (close to the p*K*_a_ value of the –NH_2_ group). That is the reason why the ability of CS to open the tight junction is confined to the duodenum. There is a need to widen the range of CS applications in the GI tract to enhance its efficacy. For this purpose, different researchers tried chemical modifications of CS [68,71,72,73]. 

Chemical modification of CS is required to improve its solubility and some other properties like hydrophilicity, mechanical properties, and ionic nature (to make it polyampholytic) and widen its applications. The reactive sites at CS for chemical modifications are (i) –NH_2_ group at C-2, (ii) hydroxyl group at C-3 and (iii) hydroxyl group at C-6. C-2 and C-6 positions are more susceptible for substitutions and grafting reactions since these are more reactive sites whereas at C-3, there is steric hindrance making it less reactive. Substitution of an alkyl group or carboxymethyl or succinyl group increases the solubility drastically. The chemical structures of *O*-carboxymethyl CS and *N*-succinyl CS are shown in Figure 5d,e, respectively. Grafting of CS improves other properties like antibacterial, anti-oxidant, chelating effect, bacteriostatic and adsorption properties [74]. To resolve the solubility problem of CS in water, *O*-carboxymethylation of CS was one of the effective attempts. *O*-carboxymethyl CS is water soluble and has high capacity to bind Ca^2+^ [75] due to the presence of the carboxymethyl group depriving the extra cellular matrix from Ca^2+^. This results in an increased paracellular permeability of the epithelium [73,76].

#### 2.1.2. Guar Gum

Guar gum is a non-ionic polysaccharide and is composed of galactose and mannose units. Its main chain consists of β-d-mannopyranose (β-d-mannose) units connected through 1,4-glycosidic linkages having side branches of β-d-galactopyranose (β-d-galactose) at every alternating mannose unit connected through 1,6-glycosidic linkages to the main backbone. The chemical structure of guar gum is shown in Figure 5f. Guar gum is used in different pharmaceutical formulations as stabilizing agent, binder, thickening agent, disintegrant, and suspending agent [15]. Guar gum finds its application in pharmaceuticals owing to its very important properties like biodegradability, non-toxicity, easy availability, and its hydrophilic nature. The hydrophilic nature of guar gum can be exploited for oral and colon specific drug delivery due to its stability over a wide pH range, controlled drug release, and more importantly the fact that it undergoes microbial degradation in intestinal fluids [36].

The most important attribute of guar gum is its susceptibility to microbial degradation in the large intestine especially when it has acidic pendant groups which facilitate its swelling at pH 7.4 in the intestine [36,77]. Wong et al. prepared 60.5% *w*/*w* guar gum matrix tablets containing dexamethasone and budesonide and studied the drug release behavior in physiological fluids. There was negligible drug release in simulated gastric and intestinal fluids whereas a considerable increase in drug release in simulated colonic fluids was observed. The tablet dissolution phenomenon was accelerated by the presence of galactomannanase enzyme and the tablets dissolution depends upon the enzyme concentration [78]. One of the derivatives of guar gum is guar gum succinate which is used for colon targeted drug delivery due to its properties such as hydrophilic nature, pH sensitivity, and the ability to retard drug release and to undergo microbial degradation.

**Figure 6:**
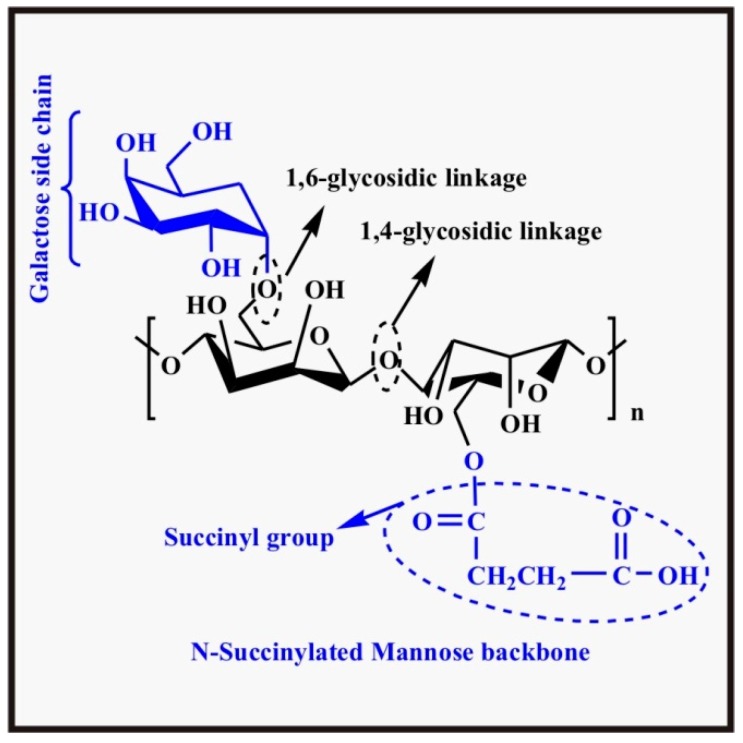
Structure of guar gum succinate.

The chemical structure of guar gum succinate is shown in Figure 6. Seeli et al. prepared guar gum succinate microparticles as a pH sensitive colon specific drug (model drug, Ibuprofen) carrier. They observed behavior of these particles in physiological fluids and found higher swelling and greater drug release at pH 7.4 SIF than at pH 1.2 SGF [36].

#### 2.1.3. Carrageenan

**Figure 7:**
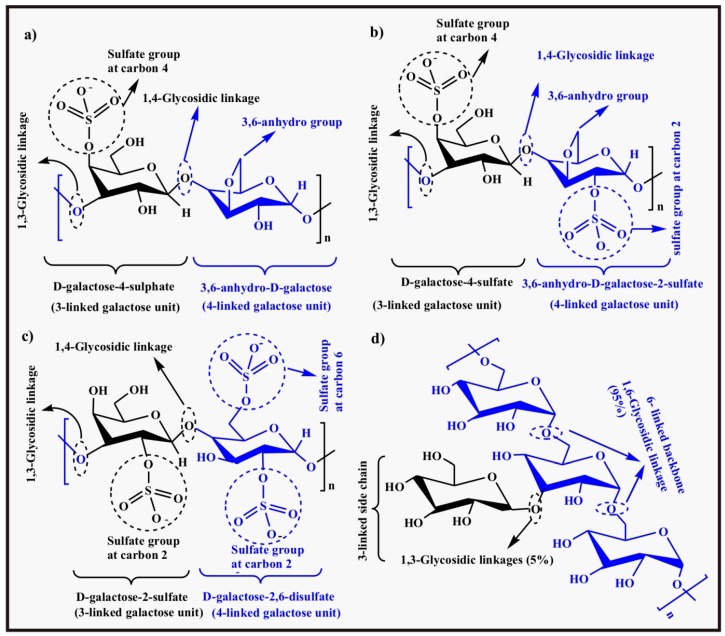
Structure of (**a**) Kappa-carrageenan, (**b**) Iota-Carrageenan, (**c**) Lambda-carrageenan, (**d**) dextran.

The word carrageenan is derived from the Irish name for the red seaweed, carrageen (meaning little rock). Carrageenan is the family name of red seaweeds (different species of Rhodophyta) from which it is extracted as it is the main component of the cell wall of red seaweeds. Carrageenan has been categorized into six different subtypes (i) Kappa, к, (ii) Lambda, λ, (iii) Nu, ν, (iv) Iota, ι, (v) Theta, θ, (vi) Mu, μ. Carrageenan is a polysaccharide made up of alternating β-d-galactose and α-d-galactose or 3,6-anhydro-α-d-galactose units and linked through α-1,3 and β-1,4-glycosidic linkages. These units may be sulfated or non-sulfated and dimers of two consecutive alternating units serve as the repeating units. The three main important commercial forms Kappa-carrageenan, Iota-carrageenan, and Lambda-carrageenan differ from one another with respect to the number of sulfate groups containing one, two and three sulfate groups, respectively on the dimer repeating units (disaccharide). The chemical structures of Kappa-carrageenan, Iota-carrageenan, and Lambda-carrageenan are shown in Figure 7a–c, respectively. 

Two forms Mu (μ), and Nu (ν) carrageenan are biological precursors of к-carrageenan and ι-carrageenan, respectively whereas, θ-carrageenan is biologically formed from λ-carrageenan. All carrageenans are water soluble but insoluble in organic solvents. The solubility in water, viscosity, and gel strength of carrageenan are controlled by the number of sulfate substituents and the equilibrium of associated cations (like Na^+^, K^+^) [79,80].

The anionic nature of carrageenan facilitates its use in pH sensitive hydrogels for controlled drug released systems. Piyakulawat et al. prepared chitosan/carrageenan hydrogel beads cross-linked with glutaraldehyde to study the controlled release of diclofenac sodium in simulated physiological fluids. They studied different compositions and found chitosan/carrageenan in a 2:1 ratio with 5% loaded diclofenac sodium as an optimized formulation. The optimized formulation was able to prolong the release up to 8 h for uncross-linked beads whereas cross-linked beads succeeded in delaying the release up to 24 h. The drug release rate was found to increase with the increase in pH of the medium, slow in SGF (pH 1.2), more in phosphate buffer (pH 6.6), and maximum release was observed in SIF (pH 7.4) because at this pH, the drug was in its completely soluble form (salt form) as well as the fact that anionic carrageenan facilitates swelling in slightly basic medium [81].

#### 2.1.4. Dextran

Dextran is a linear polysaccharide consisting of α-d-glucose linked through 1,6-glycosidic linkages (95%) but occasionally has 1,3-linkages (5%) as well [82]. The chemical structure of dextran is shown in Figure 7d. It is a well-known biocompatible and biodegradable natural polymer synthesized biologically by the bacterium *Leuconostoc mesenteroides*. The most important fact about dextran is that it is enzyme degradable by the enzyme dextranase (the enzyme that accelerates the degradation of dextran). This enzyme is present in the colon and is produced by a special kind of bacterium belonging to the genus bacteroids. This property makes dextran an excellent tool for drug delivery in the colon as dextranase degrades the α-1,6-glycosidic linkage of dextran and disintegrates the hydrogel matrix leading to targeted release of the drug [83,84]. Chiu et al. prepared pH sensitive dextran hydrogels by conjugating the activated dextran (activating agent is 4-nitrophenyl chloroformate) with 4-aminobutyric acid and cross-linking with 1,10-diaminodecane. The introduction of the carboxyl group facilitates its swelling at a higher pH of 7.4 and controls drug release in the colon region owing to the degradation of dextran in the colon as well. The release rate of BSA was studied between 2.0 and 7.4 range of pH in the presence as well as absence of enzyme dextranase [85]. Kim and Oh prepared UV irradiation cross-linked glycidyl methacrylate dextran (GMD) and poly(acrylic acid) (PAA) pH sensitive hydrogels for colon specific drug delivery. The swelling ratio of GMD/PAA hydrogels and the drug (5-aminosalicylic acid, 5-ASA) release profile studied at pH 2.0 and 7.4 in the presence and absence of dextranase. GMD/PAA hydrogels showed a high swelling ratio at pH 7.4 owing to the presence of the acidic group and it was observed that swelling was significantly enhanced nearly 45 times in the presence of dextranase. The evaluation of 5-ASA release in simulated GI pH fluids and in the presence or absence of dextranase indicates that GMD/PAA hydrogels can be a successful tool for colon targeted drug delivery [42].

#### 2.1.5. Xanthan

**Figure 8:**
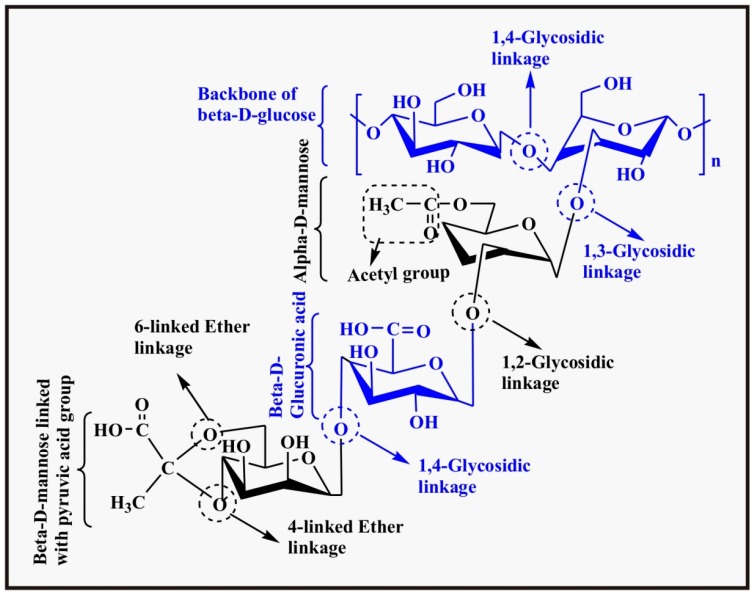
Chemical structure of Xanthan.

Xanthan is a highly branched polysaccharide with high molecular weight produced by the bacterium, *Xanthomonas compestris*, under anaerobic conditions from sugar cane. It consists of d-glucose, d-mannose, and d-glucuronic acid in a 2:2:1 ratio, a backbone of β-d-glucose (linked through 1,4-glycosidic linkage) having a trisaccharide branch connected by 1,3-glycosidic linkages on alternate glucose units of the backbone. The trisaccharide branch consists of (in sequence, backbone to last unit) α-d-mannose, β-d-glucuronic acid, β-d-mannose, and is connected (connectivity in sequence, from last unit to backbone) via β-1,4, β-1,2 and α-1,3 glycosidic linkages. Also, one acetyl group is attached to oxygen at C-6 of α-d-mannose whereas a pyruvic acid group is attached to oxygens of C-4 and C-6 of β-d-mannose via a ketal linkage [86]. The chemical structure of xanthan is shown in Figure 8.

Xanthan, being an ionic polymer, containing a carboxylic group on one of the side chain of glucuronic acid is pH sensitive and shows much swelling in basic environment due to complete ionization of the carboxylic group. The p*K*_a_ value of the carboxylic acid group is 4.6 and below this value it remains unionized and neutral whereas above this value it is ionized and carries a negative charge responsible for swelling. Hence, it can be used as a pH sensitive hydrogel for loading and controlled release of drugs in the intestinal region (pH 7.4). Bueno et al. studied the controlled release of BSA from xanthan hydrogels in the presence as well as the absence of citric acid. It was observed that BSA release is maximum at neutral and basic media due to the ionization of the carboxylic acid group and the negative charge on the hydrogel which causes swelling [87].

#### 2.1.6. Cellulose

**Figure 9:**
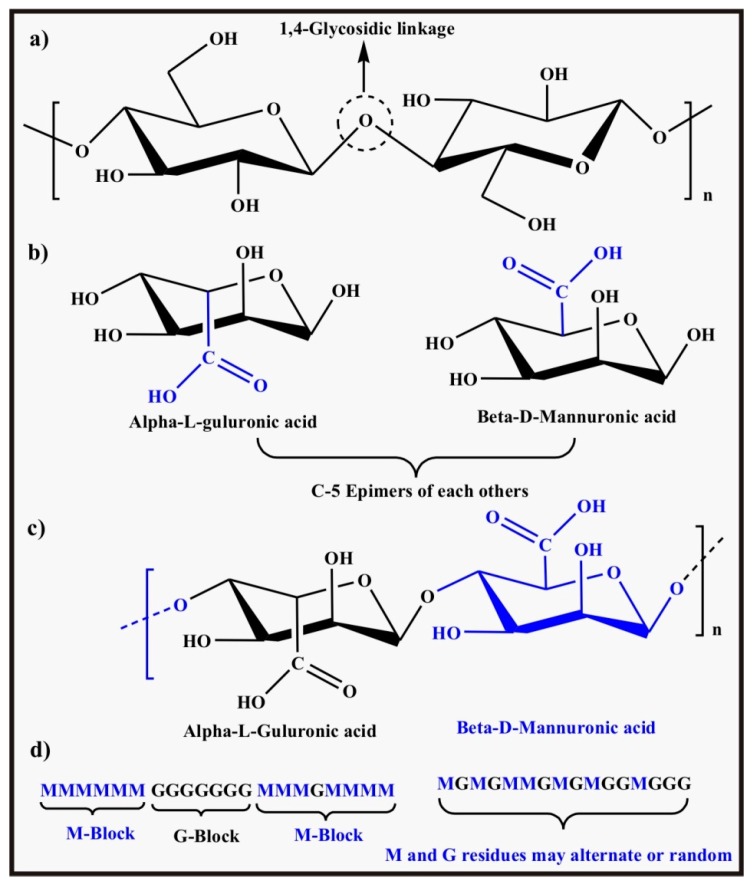
Structures of (**a**) cellulose (**b**) alpha-l-guluronic acid and beta-d-mannuronic acid (the epimers), (**c**) alginic acid (so called alginate), (**d**) arrangements of M and G residues as M and G blocks.

Cellulose is a naturally occurring polysaccharide of β-d-glucose linked through 1,4-glycosidic linkages to form linear chains. The chemical structure of cellulose is shown in Figure 9a. It is the most abundant polymer found in plants (called plants cellulose, PC) as well as being biologically synthesized in microorganisms or bacterium, e.g., *Acetobactor xylinum* (called bacterial cellulose, BC). BC has some advantages over PC such as (i) BC is totally pure whereas PC is accompanied by some other plant materials such as lignin and pectin, and needs further purification (ii) BC is more crystalline (>60%) than PC (40–60%), (iii) BC has different physical properties and macromolecular structure to PC but both are chemically similar, and (iv) BC fibers are ultra-fine, nanosized, roughly two orders of magnitude smaller than PC fibers and with more water holding capacity and greater tensile strength. 

The properties like excellent biocompatibility, biodegradability, low cost and low toxicity of cellulose and its derivatives (cellulosics) have made them an attractive choice for biomedical devices and other biomedical applications. Cellulose itself is insoluble in water but its derivatives like carboxymethyl cellulose or its sodium salt is water soluble and is suitable for pH sensitive hydrogel formation [88]. Also, many cellulose composite hydrogels with other synthetic materials have been synthesized and are observed to be successful in pH sensitive delivery of different drugs in physiological media. Lim et al. prepared cellulose nanocrystals/poly(acrylamide) hydrogels with excellent pH sensitivity and showed maximum swelling at pH 7 [89]. Zhou et al. synthesized cellulose based temperature/pH sensitive hydrogels for controlled oral drug delivery of model drug, methylene blue/methylene orange. The as-prepared hydrogels exhibited excellent pH sensitivity and showed swelling at pH 7.4 [90].

#### 2.1.7. Alginate

Alginic acid, also referred to as alginate, is an anionic natural copolymer of two stereo-isomers (i) β-d-mannuronic acid (M), and (ii) its C-5 epimer α-l-guluronic acid (G) [91]. The term C-5 epimer refers to the two stereoisomers (anomers) which differ with respect to the position of the carboxylic group on either side of the ring at carbon 5. The copolymer may be in the form of M blocks and G blocks or alternating M and G blocks or alternating/random M and G residues. Among the important sources of this natural copolymer are some algae like kelp and exopolysaccharides of bacteria like *Pseudomonas aeruginosa*. The chemical structures of the monomers, alginate, and the arrangement of G and M blocks are shown in Figure 9b–d, respectively. Alginate is water soluble and the solubility is controlled by different factors (i) ionic strength of the medium which affects properties of solution like viscosity, chain extension, conformation and ultimately the solubility, (ii) pH of solvent which should be above pH 3.0–3.5 where the carboxylic group is ionized because in the protonated form of the carboxylic group, alginate is not completely soluble in any solvent due to intermolecular hydrogen bonding leading to increased viscosity, (iii) cross-linking divalent cations like Ca^2+^, Sr^2+^, etc. which decrease the solubility (a solvent free from these ions is required to dissolve it completely). The tetrabutylammonium salt of alginate is completely soluble in organic solvents like ethylene glycol as well as in water [92]. Low cost, low toxicity, biocompatibility, and biodegradability are the attributes of alginate making it a suitable candidate for applications in the biomedical field [93]. 

The anionic nature of alginate, its derivatives, its blends, and its polyelectrolyte complexes makes it a suitable carrier for pH sensitive drug release for different drugs. Jao et al. loaded ampicillin in calcium alginate hydrogels and studied the mechanism of controlled release of the drug in simulated physiological fluids. The study of drug release in SIF (pH 7.4) and SGF (pH 1.2) showed that the drug release is pH dependent and negligible release in SGF while maximum release in SIF was observed [94]. Chen et al. blended alginate with *N*,*O*-carboxymethyl chitosan (NOCC) cross-linked with genipin and studied pH dependent release behavior of the loaded model protein drug, BSA. The swelling and drug release behavior of BSA loaded NOCC/alginate blend was evaluated in simulated physiological fluids. The results showed that only 20% of the drug was released in SGF (pH 1.2) with extensive swelling and the remaining 80% of the drug was released in SIF (pH 7.4), thus showing pH dependent control release. At pH 1.2, the NOCC/alginate blend hydrogel swells to a very small extent because the acid groups in the blend remain unionized and contribute to tight packing due to intermolecular hydrogen bonding whereas, at pH 7.4, the blend swells extensively due to ionization of acid pendant group causing severe repulsion [95].

### 2.2. Synthetic Hydrogels

#### 2.2.1. Poly(acrylic acid)

**Figure 10:**
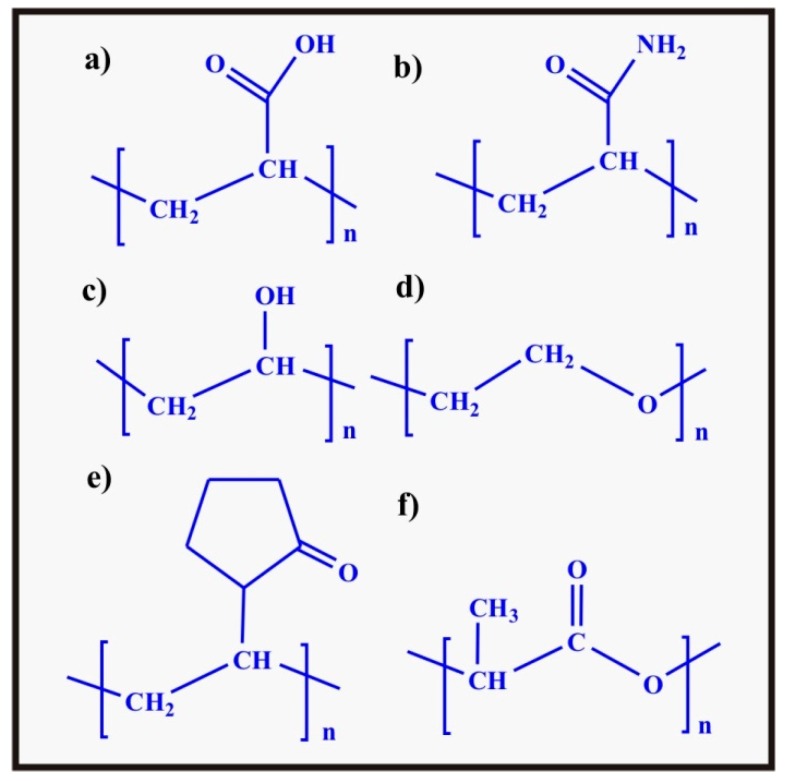
Structures of (**a**) poly(acrylic acid), (**b**) poly(acrylamide), (**c**) poly(vinyl alcohol), (**d**) poly(ethylene glycol), (**e**) poly(vinyl pyrrolidone), (**f**) poly(lactic acid).

Poly(acrylic acid) (PAA) is a commercially available, pH and electrically [96] sensitive synthetic polymer having anionic/acidic (–COOH) pendant groups on the polymer chains. The chemical structure of PAA is shown in Figure 10a. It is a potential material for biomedical applications especially for pH sensitive hydrogel based drug delivery system [89]. Pure PAA is highly soluble in water and may dissolve before drug release reflecting its fragile nature [97]. This is the reason why, acrylic acid monomers are copolymerized with some other monomers containing specific functional groups such as 2-hydroxymethacrylate, *N*,*N*′-methylenebisacrylamide, and *N*-isopropylacrylamide or cross-linked readily with poly(vinyl alcohol) and poly(ethylene glycol) by free radical polymerization or can be grafted on chains of polysaccharides [98]. The presence of an acidic group on the PAA chains makes it a suitable candidate for use as a pH sensitive hydrogel for drug delivery. The p*K*_a_ of PAA is between 4.5 and 5.0 and at pH > 5, acidic moieties become ionized developing a negative charge on the pendant groups of the polymer chains. This negative charge causes severe repulsion between the chains and eventually is responsible for extensive swelling. However, it has a minimum tendency to swell below pH 4 (stomach pH). Due to this property, pristine PAA, blended PAA [89] or PAA grafted with natural polymers [99] can be used as a successful pH sensitive hydrogel drug carrier for sustained intestinal drug delivery system [100]. Sheikh et al. prepared PAA pH sensitive hydrogels by passing an electron beam through an aqueous solution of PAA. The PAA hydrogels showed remarkable high swelling at pH > p*K*a showing their pH dependence and ability to be used as carriers for pH sensitive controlled drug delivery [101]. Chang utilized grafted starch acrylic acid monomer in the presence of *N*,*N*-methylene bisacrylamide as a cross-linking agent and ammonium persulfate as an initiator to make acrylic acid grafted starch (St-*g*-PAA) superabsorbent. Then the superabsorbent (St-*g*-PAA) was blended with sodium alginate (SA), cross-linked with Ca^2+^ to make pH sensitive hydrogels, and loaded with diclofenac sodium (DS). These hydrogels showed controlled drug release at pH 7.4 representing their pH sensitivity and controlled release in the intestine [99].

#### 2.2.2. Poly(acrylamide)

Poly(acrylamide) has its origin as old as 1959 when it was introduced as a matrix for electrophoresis [102]. Poly(acrylamide) is a pH sensitive, biocompatible synthetic polymer with good muco-adhesive properties. The chemical structure of poly(acrylamide) is shown in Figure 10b. It has been utilized in different biomedical applications especially for the delivery of drugs and proteins (insulin). Acrylamide/methacrylamide monomer has been grafted onto many natural polymers to develop different properties such as mechanical strength, pH sensitivity, muco-adhesivity, and superabsorption property in the hydrogel matrix [103]. Mukhopadhyay et al. reported pH sensitive poly(acrylamide) grafted *N*-succinyl chitosan (PAA/S-CS) hydrogels as a successful carrier for the oral drug delivery of insulin. They observed 38% insulin loading efficiency and 76% encapsulation efficiency whereas 26% insulin release was found at pH 1.2 (acidic stomach pH) and 98% release at pH 7.4 (intestinal pH). They found no toxicity in the reported hydrogels and 4.43% bioavailability. These hydrogels appeared to be successful carriers for insulin capable of lowering the blood sugar level in diabetic mice [104].

#### 2.2.3. Poly(vinyl alcohol)

Poly(vinyl alcohol) (PVA) is the first ever formaldehyde cross-linked hydrogel used for biomedical implants (synthesized in 1949) and later on it was studied for different biomedical applications such as scaffold for bone tissue regeneration, vascular prosthesis, articular cartilage replacement, skin replacement, treatment of tuberculosis etc. [3]. PVA is a synthetic, water soluble, non-toxic [105], hydrophilic, biocompatible, biodegradable [106], non-carcinogenic [107] polymer with good mechanical properties. Due to the above mentioned positive attributes, it is being used in different biomedical applications [108] such as tissue engineering and drug delivery [109]. The chemical structure of PVA is shown in Figure 10c. It has significant applications in the medical field such as drug delivery, implantable medical devices, hemodialysis, artificial pancreas as well as in some other fields like food packaging and polymer recycling [106]. It is used as a blending agent with different natural polymers to enhance its mechanical properties such as tensile strength and flexibility of blends. 

Atif et al. prepared pH sensitive chitosan and PVA (CS/PVA) blended hydrogels cross-linked with tetra-ethoxy orthosilicate to observe the dexamethasone loading and release profile in SGF and SIF. PVA has an effect on the degree of swelling (DS) of hydrogels as DS decreases with the increase in its percentage in the hydrogels. The most important point is the pH sensitivity of these hydrogels as they show low swelling in acidic and basic media but maximum swelling at neutral pH. They observed that 9.37% of the loaded drug was released in SFG (pH 1.2) over a period of 2 h whereas the remaining 90.67% was released in SIF (pH 7.4) [110].

#### 2.2.4. Poly(ethylene glycol)

Poly(ethylene glycol) (PEG) is water soluble [111], biocompatible, and non-biodegradable with extraordinary properties such as low toxicity, immunogenicity, and protein resistance. The chemical structure of PEG is shown in Figure 10d. One of the most important attributes of PEG is that it conjugates proteins, peptides, and non-peptide drugs which are less antigenic and less immunogenic and remain stable towards degradative enzymes. PEG can be copolymerized with aliphatic esters like poly(lactic acid) to enhance the biocompatibility of the polymers followed by use in drug delivery and tissue engineering applications. Atta et al. prepared pH sensitive chitosan and poly(ethylene glycol) (different molecular weights) blended hydrogels (CS/PEG) as carriers for cefixime (a model drug). They observed that swelling in water was enhanced with increasing molecular weight of PEG. The CS/PEG hydrogels showed low swelling in neutral medium whereas maximum swelling in acidic and basic media was observed [20].

#### 2.2.5. Poly(vinyl pyrrolidone)

Poly(vinyl pyrrolidone) (PVP) is highly water soluble with excellent properties like biocompatibility and absorbancy but poor mechanical properties. However, to overcome the poor mechanical properties, PVP can be grafted, or its monomer, *N*-vinyl pyrrolidone, can be copolymerized with some other vinylic monomers such as acrylic acid, methacrylic acid, methacrylamide, etc. The chemical structure of PVP is shown in Figure 10e. Sohail et al. prepared pH sensitive poly(vinyl pyrrolidone)/acrylic acid hydrogels (PVP/AA) and studied the swelling and drug release profile of tramadol (model drug) at different pH values (pH 1.2, 5.5, and 7.5). Swelling and drug release decreased with increasing cross-linking density while there was an increase in both factors with increase in pH of the medium and acrylic acid monomer content. However, the drug release profile at different pH values showed that drug release was minimum at pH 1.2 and maximum at pH 7.5 which indicates that PVP/AA is a successful candidate for colon targeted drug delivery [96]. 

#### 2.2.6. Poly(lactic acid)

Poly(lactic acid) (PLA) is a synthetic aliphatic polyester derived from naturally occurring lactic acid and FDA approved degradable polymer. PLA is a thermoplastic, biocompatible, biodegradable, non-toxic, and eco-friendly polymer with excellent mechanical properties. The chemical structure of PLA is shown in Figure 10f. PLA and its composites are biodegradable and degrade under physiological conditions to harmless and non-toxic products that can be easily excreted through the kidneys. These attributes of PLA, its composites and its copolymers make it a strong candidate for different biological applications such as tissue engineering, drug delivery systems, and various medical implants. Recently, researchers have been focusing on the use of PLA and its copolymer poly(lactic-*co*-glycolic) acid, PLGA as novel drug carriers for localized delivery on account of their biocompatibility, biodegradabilityand easy processability [112].

### 2.3. Hybrid Hydrogels

The term hybrid hydrogels refers to a class of hydrogels in which two components belong to two distinctive classes like organic and inorganic (maybe nanoparticles) or biological polymers (natural) and synthetic polymers cross-linked physically or chemically. Mixing of such components may lead to materials with improved properties compared to both the individual components. The terms hybrid and composite hydrogels are actually synonymously used in the literature to describe the hydrogels with a mixture of natural and synthetic polymers [113] or polymer hydrogels mixed with non-hydrogel material such as inorganic/metal nanoparticles [114,115]. The hybrid of carboxymethyl chitosan and poly(ethylene oxide)/poly(propylene oxide)/poly(ethylene oxide) (PEO-PPO-PEO) cross-linked with glutaraldehyde for ophthalmic drug delivery of nepafenac is an example of a hybrid of natural and synthetic hydrogels. The controlled delivery of this model drug was observed and delivery was found to be maximum at 35 °C and pH 7.4. The hybrid hydrogel was found to be non-toxic for human corneal tissue as well, at low concentration [116]. Anjum et al. prepared hybrid hydrogels consisting of chondroitin sulfate and poly(ethylene glycol) for the delivery of growth factors and a variety of biomedical applications [117]. 

The use of hydrogels in the biomedical field and tissue engineering has been known for a long time. To improve their performance in the field of biomedicine, researchers have tried to make their composites with different metallic nanoparticles. This improves their bioactivity and mechanical properties. Zare-akbari et al. prepared bionanocomposite hydrogel beads comprised of carboxymethyl cellulose/ZnO nanoparticles hybrid (ZnO/CMC) physically cross-linked with Fe^3+^ ion, an ionic cross-linker. This represents an example of a hydrogel hybrid of natural polymer hydrogels and inorganic metal nanoparticles (the non-hydrogel). The release behavior of the loaded model drug, propranolol hydrochloride was studied and it was found that the swelling and drug release was maximum in SIF at pH 7.4 as compared to pH 1.2 in the gastric environment. This study also includes ZnO/NaCMC nanocomposite which showed more swelling and more controlled drug release than that of ZnO/CMC hybrid hydrogels which in turn showed better swelling and controlled drug release behavior than neat hydrogel [118]. Khaled et al. synthesized novel hybrid hydrogels for loading and delivery of small interfering RNA (siRNA) in acidic environment representing a class of hybrid hydrogels containing inorganic nanoparticles dispersed in an organic matrix. Hybrid hydrogels consist of fluorescently doped silica nanoparticles coated with cationic poly(2-diethylaminoethylmethacrylate) hydrogel matrix. The loaded siRNA can be released in acidic environment exploiting the acidic nature of the matrix and is responsible for the decrease in the expression of protein in human breast cancer cell line [119]. Another example of an organic (natural) and inorganic hybrid is the use of graphene oxide as a nano-drug carrier incorporated in the cationic chitosan matrix to study the release behavior of BSA as model drug. Cationic chitosan was prepared by the reaction of chitosan with glycidyltrimethylammonium chloride and was mixed with separately prepared graphene oxide-drug intercalation complex to form hybrid hydrogels. The hydrogels showed more swelling and drug release up to pH 6.8 and swelling decreased onwards up to pH 7.4 indicating their pH sensitivity [120]. 

## 3. Applications of pH Sensitive Hydrogels

**Figure 11:**
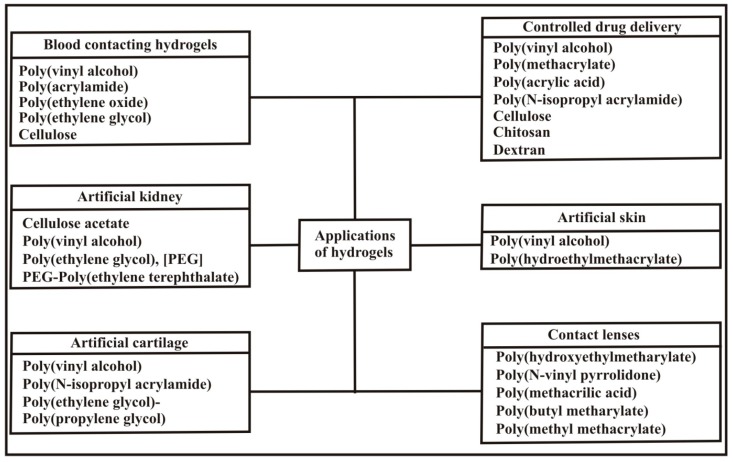
Applications of hydrogels in different biomedical fields.

Amongst stimuli sensitive hydrogels, pH sensitive hydrogels have been widely studied and used in biomedical applications especially in drug delivery applications exploiting the pH variation along the GI tract. pH sensitive hydrogels find applications in different biosensors (micro devices) like BioMEMS (Biomedical microelectrochemical systems) utilizing pH sensitive hydrogels consisting of a poly(methacrylic acid) and poly(ethylene glycol) blend [121]. Applications of hydrogels in different biomedical fields are summarized in Figure 11. Aside from the use of pH sensitive hydrogels in biomedical applications, they are being effectively exploited in the engineering field as microfluidic valves [122] to control the flow of liquids owing to their swelling and deswelling in response to the pH of the flowing medium. Based on these responses, the pH of the flowing medium opens and closes the microvalve and consequently controls the flow [123,124]. The pH sensitive hydrogels, the kinds of drug loaded, and their functions are given in Table 2. 

### 3.1. Controlled Drug Delivery

**Figure 12:**
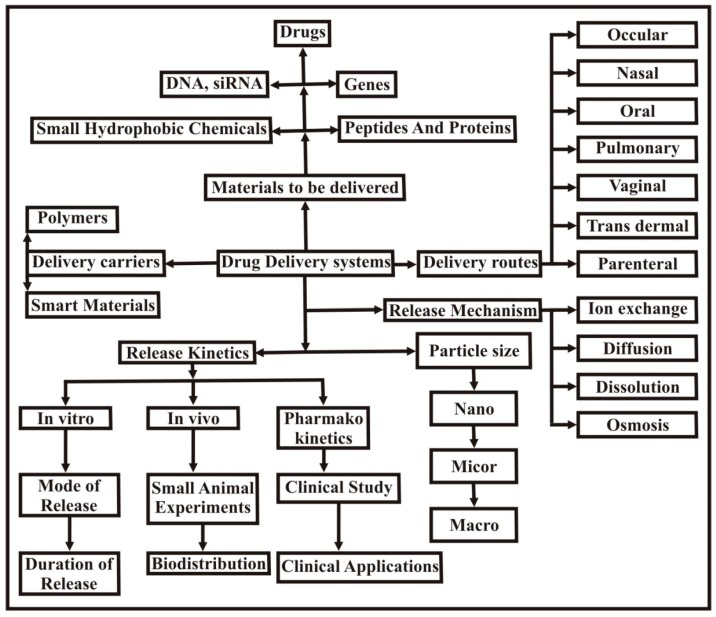
Drug delivery development from basic research to applications.

Currently, researchers are focusing on improving the efficacy ratio of existing drugs due to the high cost and extensive time involved in the synthesis of new drugs [137]. For this purpose various drug delivery systems have been developed and are shown in Figure 12. 

The attributes of ideal drug delivery systems include biocompatibility, biodegradability, able to load the drug in high amounts, and release it in the required controlled manner. The most common drug delivery formulations are sustained release, delayed release, and repeat release/action formulations. An enteric coated tablet does not release the drug at all in the acidic stomach environment but releases the amount of drug in the neutral/slightly basic intestine environment which is referred to as delayed release. Whereas, in the case of sustained release drug delivery systems, the drug is entrapped in the carrier matrix and a small portion of it is released in the stomach and the remaining portion is released in a controlled manner in the intestine. In repeated release or fixed dosage combinations, there is immediate release followed by sustained release of the same drug or two different drugs [138]. Oral drug delivery is the main route for delivering the therapeutics to selected sites owing to the sensitivity of the carrier to specific stimulus like temperature, pH, etc. Since, there is a wide pH gradient change along the GI tract with salivary pH of 6.7–7.3 in the mouth (closer to 7.5 for a healthy individual, an alkaline pH that protects enamel from acidic corrosion) [139] and ranging from the acidic pH of the stomach (pH 1.0–3.0) to the alkaline pH in the intestine (pH 5.0–8.0), this variation can be exploited as a stimulus for pH sensitive hydrogel carriers for drug delivery applications. Some other diseased parts of the body have different pHs such as the pH of chronic wounds ranges between 5.4 and 7.4 and that of extracellular matrix in cancer tissue is acidic [35]. The pHs of different parts of the alimentary canal and some other tissues and organelles are tabulated in Table 3. 

### 3.2. Drug Delivery in the Stomach

Drugs that do not exhibit homogeneous absorption (narrow absorption window) throughout the GI tract such as para-amino benzoic acid and those which have poor absorption at higher pH such as Verapamil HCl are particularly important for delivery in the stomach [138,140]. Localized drug release in the stomach is extremely significant for the treatment of gastric cancer, gastritis, carcinoma [143], and gastro duodenal ulcer using chitosan based hydrogels. *Helicobacter Pylori* (*H. Pylori*) which inhabits the mucous layer adhering to gastric epithelial cells, is responsible for the above mentioned gastric diseases which are treated with amoxicillin, metronidazole, and clarithromycin. Therapies using a single antibiotic may not appear successful due to inadequate permeation of the drug across the gastric mucous layer and its lower stability in acidic medium. To increase the drug resident time in the stomach and its sustained release, chitosan, its derivatives and its blends are used as successful carriers [144]. Kumar et al. prepared pH sensitive hydrogels for stomach targeted drug delivery of clarithromycin. Acrylic acid was used to graft chitosan by free radical polymerization using ammonium persulfate as initiator, blended with poly(vinyl pyrrolidone) and cross-linked using glutaraldehyde and *N*,*N*′-methylenebisacrylamide as cross-linker. They observed that these covalently cross-linked hydrogels were pH sensitive and showed maximum swelling and drug release at low pH in the stomach environment owing to protonation of an amino group achieving the targeted sustained release. The purpose of introducing acrylic acid as graft is to control the swelling (to avoid burst release) and to achieve sustained release of the antibiotic [125]. 

El-Mahrouk et al. prepared pH sensitive chitosan based hydrogels cross-linked with tripolyphosphate (TPP) loading metronidazole for the eradication of *H. Pylori* from the stomach. The prepared hydrogels showed a higher degree of swelling and a greater amount of drug release at gastric (acidic) pH than at intestinal (neutral) pH. The results showed that the retention of these hydrogels in a dog’s stomach was up to 48 h (sustained enough to eradicate *H. Pylori*) exhibiting more efficacy than commercially available metronidazole tablets [126]. Risbud et al. prepared pH sensitive interpenetrating hydrogels composed of chitosan (CS) and poly(vinyl pyrrolidone), (PVP) cross-linked with glutaraldehyde for stomach targeted release of the antibiotic (amoxicilin). The prepared freeze-dried membranes showed more swelling and better drug release (73%) at pH 1 in 3 h compared to air dried membranes. Freeze dried membranes appeared to be better candidates for stomach targeted controlled drug delivery of antibiotics for the eradication of *H. pylori* [127]. 

### 3.3. Drug Delivery in the Intestine

Orally taken drugs are mainly absorbed in the intestine and the main target of oral drug delivery is to transport the drugs from the acidic stomach to the weakly basic intestine safely while maintaining the bioactivity of the drugs (low acidic pH in the stomach environment degrades proteins and peptides). Other problems that drugs face during their transportation through the varying pHs along the GI tract, pH 1.2 (stomach) to pH 8 (intestine) may be poor permeability across GImucosa, acid catalyzed degradation of drugs, and proteolytic degradation along the GI tract [145]. These problems are mainly resolved by avoiding drug release in the stomach using such hydrogel carriers that remain shrunk in the stomach acidic environment so that the drug cannot be released. For this purpose, natural polymers with anionic pendant groups like –COOH/–SO_3_H are selected which remain protonated in acidic medium. As a result, hydrogels retain their shrunk state and avoid drug release in this acidic environment. Acrylic acid and its derivatives are usually used to graft natural polymers to impart pH sensitivity to achieve the target of transporting the drug through the acidic stomach as safely as possible to the intestine where maximum drug is released and also absorbed [129]. Sun et al. prepared pH sensitive hemicelluloses-*co*-acrylic acid grafted hydrogels cross-linked with *N*,*N*′-methylenebisacrylamide. They studied theophylline and acetylsalicylic acid as a model drug and found that the prepared hydrogels exhibited negligible swelling and drug release in SGF (pH 1.2) and maximum swelling and drug release (85%) in SIF (pH 7.4). This swelling and drug release behavior was attributed to the ionization of the carboxylic group on the grafted acrylic moiety which ionizes above its p*K*_a_ value (p*K*_a_ = 4.28) [97]. The ionized carboxyl groups, –COO^−^ on the adjacent chains repel each other causing swelling and drug release at pH 7.4 [128]. 

Some drugs have special requirements for their delivery, for example dexamethasone has a short half-life (2–5 h) in plasma, low solubility in aqueous solution, and is also used in the treatment of ulcerative colitis along with some other diseases. Due to having a short half-life and being used in the treatment of ulcerative colitis, it has to be delivered in the lower GI tract where it can be absorbed immediately and to maximum extent and is effective for local treatment of the said disease. Das et al. successfully synthesized acrylic acid grafted guar gum/β-cyclodextrin blended hydrogels cross-linked with tetraethyl orthosilicate (TEOS) for the intestine targeted delivery of dexamethasone. Dexamethasone is an anti-inflammatory, immune suppressive agent and requires delivery to be at the lower GI tract for the treatment of inflammatory bowel disease and ulcerative colitis. The prepared hydrogels showed pH sensitivity and exhibited prolonged drug release with increasing guar gum content and maximum release at high pH in the intestine (pH 7.4) [97]. Hydrophobic drugs like atorvastatin have very low solubility in aqueous medium as well as very low bioavailability which are supposed to be their limitations in oral administration. Different strategies have been employed to improve the bioavailability and solubility of hydrophobic drugs in aqueous medium such as formation of complexes with β-cyclodextrin, conversion of the crystalline state into the amorphous one, and decrease in the particle size. Yang et al. prepared dual pH and thermoresponsive β-cyclodextrin-*co*-methacrylic acid hydrogels for intestine targeted delivery of atorvastatin. These hydrogels showed maximum swelling and nearly 90% drug release in slightly alkaline medium (pH 8.06) and negligible swelling and minimum drug release at low pH. As thermoresponsive hydrogels, the swelling ratio increased with increasing temperature (30–45 °C) [129]. 

Chronic conditions of general anxiety disorder (GAD), epilepsy. and neuropathic pain are managed by introducing the sustained release of second generation anticonvulsant drugs like pregabalin (PGB). Cevik et al. introduced a novel method of visible light induced synthesis of pH sensitive hydrogels for delivery of pregabalin at intestinal pH. The hydrophobic styrene-butadiene-styrene (SBS) copolymer was incorporated into the pH responsive methacrylic acid-*g*-ethylene glycol P(MAA-*g*-EG) prepolymer for increased integrity and hydrophobic function. The prepared P(MAA-*g*-EG) hydrogels showed high swelling at high pH 7.4 and nearly 86% drug release whereas low swelling and less drug release (15.3%) at low pH. With SBS incorporated into the pH sensitive prepolymer, swelling decreased and drug release was 79.5% at pH 7.4 and only 14.9% at pH 1.2 [130]. Wang et al. prepared pH switchable hydrogels comprising of lignosulphonate grafted poly(acrylic acid)-*co*-poly(vinyl pyrrolidone)). In vitro drug release was done using amoxicilin as model drug and there was 51.5% drug release in 24 h in SGF whereas 84.5% drug release in 24 h in SIF in separate studies. The swelling and drug release was observed more at pH 7.4 than at pH 1.2 [131]. Insulin used in the treatment of diabetes suffers degradation in the acidic stomach environment during its oral administration. So, there is a need to develop a pH sensitive carrier that can safely transport insulin through the stomach into the intestine. Zhang et al. developed pH sensitive chitosan based hydrogels for the safe oral delivery of insulin. They prepared pH sensitive hydrogels by grafting acrylic acid onto the chains of carboxymethyl chitosan loaded with insulin. In vitro studies showed that 93% drug was released in SIF while only 16.3% drug was released in SGF. In vivo animal studies showed an effective and persistent hypoglycemic effect [132].

### 3.4. Drug Delivery in the Colon

For poorly absorbed drugs in the upper GI tract and digestion problem of proteins and peptides in the acidic stomach environment, researchers have found a novel drug delivery mode in the colonic region where drugs are absorbed through systematic circulation. Colonic drug delivery is useful for treatment of arthritis, angina, nocturnal asthma and especially for the localized treatment of colonic diseases like colorectal cancer, Crohn’s disease, and ulcerative colitis [137]. Colorectal cancer is usually treated by surgery, chemotherapy, radiation therapy, and biological therapy. The treatment to be selected depends upon the stage of cancer and combination of two or more treatments may be applied simultaneously [146]. Ghaffar et al. prepared pH sensitive acrylic acid grafted starch hydrogels to study the controlled release of rutin in the lower GI tract (colon). The prepared hydrogels exhibited a higher degree of swelling as well as more drug release at pH 7.4 than at pH 1.2. This behavior is attributed to the presence of an acidic carboxylic group which ionizes closer to neutral and at alkaline pH to give anionic pendant groups which are responsible for the swelling. Histopathological testing of these hydrogels showed release of rutin in negligible quantity before the colon and controlled delivery in the colon [133].

pH sensitive hydrogels susceptible to microbial degradation exhibit an additional feature for controlled and sustained drug release in the colon. Guar gum and its derivative based composite hydrogels are one of the sources for retarded drug release in the large intestine accompanied by the inherent microbial degradation of guar gum based hydrogels. Seeli et al. prepared pH sensitive hydrogels beads composed of guar gum succinate-sodium alginate (GGS-SA) for the study of the controlled release of ibuprofen. The observed behavior of the hydrogel beads showed more swelling and drug release at pH 7.4 than at pH 1.2 which is attributed to the presence of anionic groups on the chains of SA while the controlled drug release is due to the presence of guar gum succinate [134]. Wang et al. prepared pH sensitive sodium alginate hydrogels cross-linked with calcium chloride to control the release of hydrocortisone from sodium succinate targeted in the colon. Due to the presence of carboxyl groups on the alginate chains, this composition has the ability to switch its response with pH reflecting it to be a strong candidate for targeted drug delivery in the colon. The prepared hydrogels showed a higher degree of swelling and more drug release at higher pH than at low pH. The incorporation of glycerol monoglyceride in the alginate helps to prolong the drug release [135]. Anirudhan et al. synthesized pH switchable hydrogels for colon targeted drug delivery to treat colon cancer. These hydrogels are composed of β-cyclodextrin grafted gelatin cross-linked with oxidized dextrin and observed the control release of an anticancer drug, 5-fluorouracil. The drug release profile studies reflected greater release in SIF, pH 7.4 than in SGF, pH 1.2. In comparison to neat drug, drug loaded hydrogels represented significantly improved cell inhibition for colon cancer cells during an in vitro cytotoxicity study [136].

### 3.5. Delivery of Proteins

#### 3.5.1. Delivery of Insulin

Even after 92 years of discovery, insulin faces lots of challenges in its widely accepted way of oral delivery. Oral delivery of insulin is accompanied by different advantages like rapid hepatic insulinization, prevention of peripheral hyperinsulinemia, weight gain, hypoglycemia, and higher patient compliance [147]. However, there are disadvantages as well; being protein in nature, insulin undergoes degradation by enzymes in acidic milieu stomach and its permeability across the intestinal wall is very poor. To overcome these problems, various drug delivery strategies have been designed such as pH sensitive hydrogels, microparticles, nanoparticles, permeation enhancers, and insulin conjugates [148]. The reason why oral delivery of insulin is preferred over injection is that injections may cause lipoatrophy and lipohypertrophy. Through injections, insulin enters the blood circulation directly causing peripheral hyperinsulinemia leading to hypoglycemia, cancer, atherosclerosisand peripheral hypertension [149]. Many researchers have tried pH sensitive hydrogels for oral delivery of insulin to avoid its degradation in the acidic environment of the stomach utilizing the inherent nature of anionic hydrogels that only swell in the slightly alkaline medium of the intestine. Rasool et al. synthesized acrylic acid grafted kappa carrageenan pH sensitive hydrogels cross-linked with vinyl triethoxysilane, a novel cross-linker. They observed the in vitro drug release profile of insulin and found it to be a controlled release in SIF with negligible release in SGF. This hydrogel is considered successful for insulin delivery as it is also hydrolyzed within 6 h after the release of insulin [38]. Demirdirek et al. developed pH sensitive poly(anhydride-ester) and poly(acrylic acid) based hydrogels for the controlled release of insulin and salicylic acid for diabetic patients. From the drug release profiles, it was observed that hydrogels released 4–8% insulin in acidic milieu (SGF, pH 1.2) and 70% salicylic acid and 90% insulin in SIF (slightly basic milieu). The results showed that the prepared hydrogels are a successful synthesis for pH dependent delivery of drugs [150].

#### 3.5.2. Delivery of Bovine Serum Albumin

BSA is one of the most abundant proteins in vertebrates and is cheap. Being a protein in nature, it degrades under acidic milieu (especially low pH resembling stomach pH) very quickly. Its oral administration makes it difficult to avoid the low pH of the stomach where it can be degraded easily. So, there is need to develop such a carrier that can keep it protected in the stomach environment and can release it safely in the intestine where it can be absorbed easily. For this purpose, pH sensitive hydrogels carrying anionic pendant groups are the best choice for the delivery of such a sensitive protein. Durgonuv et al. prepared gamma irradiated pH sensitive hydrogels composed of chitosan and poly(vinyl pyrrolidone) in different compositions for the safe delivery of BSA. These hydrogels showed maximum adsorption of BSA at pH 5.0 and maximum release at pH 7.4. The adsorption capacity of as-prepared hydrogels has been observed to increase from 0 to 350 mg BSA per gram dry hydrogels [151]. Gao et al. prepared pH sensitive poly(acrylic acid) hydrogels by cross-linking with poly(glutamic acid)-*g*-(2-hydroxy methacrylamide)) for the controlled release of BSA. The prepared hydrogels showed greater swelling in the slightly alkaline medium of the intestine with much less swelling in acidic medium resembling the stomach. The purpose of protection of BSA from the acidic degradative environment of the stomach seems to be achieved by these hydrogels since the release of the loaded BSA was maximum in neutral medium and minimum in acidic medium [152]. Yang et al. prepared pH sensitive hydrogels by grafting methoxy poly(ethylene glycol) onto carboxymethyl chitosan (mPEG-*g*-CMC) and alginate for the delivery of BSA. They also compared the properties of mPEG-*g*-CMC hydrogels with that of the blend of mPEG and CMC. The swelling behavior and BSA release was less in acidic medium (pH 1.2) than that in slightly alkaline medium resembling that of the intestine. Burst release at pH 1.2 was decreased by loading BSA in mPEG-*g*-CMC/alginate hydrogels [153].

### 3.6. Delivery of Genes

The delivery of genes/DNA requires a carrier system that can protect the genes against the degradation and aggregation in the extracellular matrix and can continuously supply it to the targeted cells. Thus, in contrast to conventional delivery systems, a controlled delivery system with better performance is required which is a combination of a hydrogel substrate and gene vector. Hu et al. synthesized temperature and pH sensitive hydrogels composed of poly(*N*-isopropylacrylamide)-*co*-poly(acrylic acid)-*co*-poly(caprolactone) hydrogels for intelligent gene delivery systems. These hydrogels were used to immobilize DNA plasmid which was compacted with heparin modified poly(ethylene imide). The low content of poly(caprolactone) in hydrogels was found to be efficient for immobilization of DNA complexes. pH dependent swelling studies showed that these hydrogels swell more at alkaline pH and less at low pH which is attributed to the presence of carboxylic group moieties of acrylic acid. Also, swelling was affected by the presence of poly(caprolactone) and the swelling decreases with increasing concentration. The DNA release kinetics study showed that hydrogels with higher contents of poly(caprolactone) exhibited faster release and the slowest release was shown by the hydrogels with no poly(caprolactone). On the other hand, the DNA release was faster with low acrylic acid content in the hydrogels due to weak electrostatic interactions [154]. Li et al. prepared multifunctional gene carriers utilizing a layer-by-layer technique with the top layer being a pH sensitive hydrogel for efficient gene delivery. The cationic core acting as template was made by condensing deoxyribonucleic acid (DNA) with protamine to give DNA/protamine complexes. This first layer was covered with subsequent layers of anionic DNA, cationic liposomes and finally the *O*-carboxymethyl chitosan (CMCS-CLDPD complexes) involving a layer-by-layer technique. The topmost layer of the CMCS hydrogel helps to enhance gene transfection efficiency and protects the CMCS-CLDPD from interaction with serum. In vivo and in vitro studies showed that the CMCS layer fell off in the tumor environment at pH 6.5 and this helped the loaded DNA to be released more quickly in the acidic medium of the tumor than in neutral medium [155].

### 3.7. Drug Delivery via Injectable Hydrogels

Drug transportation using injectable hydrogels has obtained greater significance for localized and prolonged delivery of drugs in a single administration. This will minimize the side effects and systematic drug concentration [156]. The sites of ischemia, tumor, and wound healing are found to be acidic. So, dual pH and temperature sensitive hydrogels have attracted researchers to develop drug delivery systems that can target regions of local acidosis [157]. For this purpose, drug carrying pH responsive hydrogels should respond by swelling in the region of local acidosis (pH < 7 down to 5) to release the required drug in a controlled manner and should show very low swelling at around blood pH (7.40). Garben et al. synthesized pH sensitive hydrogels composed of copolymer of *N*-isopropylacrylamide and propylacrylic acid for growth factor controlled release as injectable hydrogels. These hydrogels have the potential to release the growth factor over a period of three weeks [157]. One of the most important characteristics of injectable dual pH/temperature sensitive hydrogels is their ionic nature which leads to the formation of an ionic complex between some bioactive species/factors and the hydrogels. The hydrogels with positively charged pendant groups can form an ionic complex with anionic species like insulin whereas negatively charged hydrogels form a complex with cationic species like transforming growth factor beta. Huynh et al. synthesized a novel class of amphoteric hydrogels (poly(urethane-amino-sulfamethazine) based block copolymer) that can form cationic and anionic hydrogels in response to acidic and basic pH, respectively. The aqueous solution of these copolymers exhibits a gel form in the 6.8 to 8.2 pH range with increasing temperature whereas a free flowing sol form in slightly acidic and basic media. These amphoteric hydrogels were tested for the release of anionic protein and human growth hormone (hGH), and their release was found to be very much controlled within three days (after the injection) with least initial burst release in Sprague-Dawley rats’ serum as compared to hGH solution. This controlled release of hGH by these amphoteric hydrogels is attributed to the ionic complex formation between cationic species of the hydrogels and negatively charged protein [158].

### 3.8. Trans Dermal Drug Delivery (pH Sensitive)

The topmost layer of skin is biologically termed stratum corneum and its structural features such as cohesion, intercellular lipid, homeostasis of stratum corneum, and permeability barrier etc. are influenced by many factors including pH of the skin. The pH of normal and healthy skin is in the range of 5.0–6.0 and this is the reason why stratum corneum is known as acid mantle. The pH of acid mantle is influenced by many factors like age, gender, sebaceous glands, apocrine glands, eccrine glands, and epidermal cells. The unbalanced pH of the skin leads to many disorders like skin illness (acne, inflammation, and irritation) as well as decreased permeability barrier and cell cohesion in the stratum corneum. When the pH of the skin layer is greater than 6.0, it leads to micellization while a pH below 4.5 results in structural disorders. Kwon et al. prepared pH sensitive hydroxyethyl cellulose/hyaluronic acid (HECHA) composite hydrogels cross-linked with divinyl sulfone to study the controlled drug release of isoliquiritigenin (ILTG) to treat propionibacterium acnes. At pH 7, ILTG loaded HECHA13 hydrogels (containing HEC and HA in a 25:75 ratio) showed an efficiency greater than 70% and inhibited the growth of acnes with excellent skin permeability (penetration via hair follicles) [142].

Patch dermal therapy is particularly important and popular especially when prolonged administration is required to avoid side effects. Many natural polymers and their derivatives like chitosan and carboxymethyl guar gum and synthetic biocompatible polymers like poly(vinyl alcohol) have been used for the encapsulation of drugs as they form free standing membranes. Giri et al. synthesized nanosilica/acrylic acid grafted guar gum membranes for transdermal patch therapy to study the drug delivery of diclofenac. Guar gum-*g*-acrylic acid, GG-*g*-AA_10/0.1/0.5_ (subscripts indicates proportions of acrylic acid, initiator and nanosilica, respectively) showed maximum water uptake between all the compositions. GG-*g*-AA nanocomposites showed a more controlled release of diclofenac than that of neat guar gum. A drug release profile study showed that release percentage decreased with decreasing percentage of nanosilica in the nanocomposites. GG-*g*-AA_10/0.1/5.0_ containing maximum percentage of nanosilica exhibited maximum drug release capacity whereas GG-*g*-AA_10/0.1/1.0_ with the least proportion of nanosilica have the least ability to release diclofenac drug [159].

## 4. Challenges and Opportunities

Despite the fact that pH sensitive hydrogels have found applications in various fields from drug delivery to tissue engineering applications, there are still challenges for the development of a hydrogel which can behave in the desired manner under acidic and basic conditions. Also the development of hydrogels which can degrade at the required duration is highly demanded in tissue engineering applications. Similarly, swelling behavior is considered really important for hydrogels because fluid uptake is a key factor during tissue regeneration; however some hydrogels lose their mechanical strength as a result of solution absorption. 

There are many of fields where pH sensitive hydrogels have opportunities, for example, in skin tissue regeneration angiogenesis, which is the formation of new blood vessels from existing vessels essential for normal healing. For enhancing angiogenesis, pro-angiogenic agents have been found to be very effective whereby pH sensitive hydrogels would be ideal candidates for their controlled release at the wound site.

## 5. Conclusions

Biocompatibility, biodegradability, and non-toxicity are the main attributes of any material to be used for biomedical applications. Amongst all the stimuli, pH and temperature exist naturally in the internal environment of the human body. Hence, internal stimuli responsive hydrogels can be exploited for site specific drug delivery. The response time of other external stimuli (light, electric field, etc.) responsive hydrogels is very slow. That is the reason why internal stimuli responsive hydrogels with smaller size are usually preferred. The pH sensitive swelling can be engineered by grafting or copolymerizing certain anionic/acidic monomers (acrylic acid/acrylamide) or cationic groups such as quaternary ammonium groups on polymer chains and can be used for site specific drug delivery to improve the efficacy ratio. The polymers (e.g., guar gum and dextran) which are susceptible to microbial degradation can be used in modified form with pH sensitive moieties or blended with pH sensitive polymers to enhance their acidic pH resistance in stomach. The drug release mechanism in the intestine simultaneously involves diffusion as well as microbial degradation (chemical release mechanism). In this way, the peptides and proteins can be safely carried through the acidic milieu of the stomach and are released in the less proteolytic environment of the intestine where they can be absorbed easily.

In order to induce fast responsiveness in hydrogels, the size should be smaller and thinner. Unfortunately, this makes the hydrogels very fragile thus compromising their mechanical strength which is one of the major characteristics required for biomedical applications. To overcome this drawback, researchers are focusing on the synthesis of new polymers by grafting or copolymerization of monomers accompanied by the discovery of novel and non-toxic cross-linkers. These novel syntheses would lead to overall, non-toxic, biocompatible, and biodegradable hydrogels having high drug loading/encapsulation efficiency with quick response to stimulus.

## Figures and Tables

**Table 1:** Examples of different types of stimuli responsive hydrogels along with their mechanisms in brief

Nature of Stimulus	Stimulus	Mechanism	Example	Ref.
**Physical stimuli**	**Temperature**	Shift in temperature changes polymer-polymer and polymer-water interaction responsible for swelling and drug release.	Chitosan-Poly(acrylamide)	[27]
	**Pressure**	Swelling under increased pressure and vice versa. This fact is due to an increase in lower critical solution temperature (LCST) value of hydrogels with pressure. LCST is the temperature below which negative thermoresponsive hydrogels swell.	Poly(*N*-isopropylacrylamide), poly(*N*,*N*-diethylacrylamide)	[28,29]
	**Light**	Exposure to light (UV and visible light) reversibly changes the hydrogel from its flowable form to non-flowable form and vice versa.	Poly(trimethylenium iminium trifluorosulfonimide) and 2,6-bis(benzoxal-2-yl)pyridine blend	[3,30]
	**Electric field**	Changes in electrical charge distribution within the hydrogels matrix on the application of electric field cause swelling–deswelling and is consequently responsible for the on demand drug release.	Polythiophene and polypyrrole	[28,31]
	**Magnetic Field**	When a magnetic field is applied, it causes pores in the gel to swell leading to drug release.	Magnetite nanoparticles and poly(acrylamide) composite hydrogels	[32]
	**Ultrasound irradiation**	Exposure to ultrasound temporarily breaks the ionic cross-links in the hydrogels and the drug is released but cross-links are reformed on discontinuation of the ultrasound waves. This facilitates on-demand drug release.	Calcium alginate Poly(lactic acid)	[33,34]
**Chemical stimuli**	**pH**	Shift in pH causes change in the charge on the polymer chains leading to swelling and drug release.	Poly(acrylic acid), Guar gum succinate, Kappa-carrageenan/poly(vinyl alcohol)	[35,36,37]
	**Ionic strength**	Change in ion concentration also causes swelling and drug release.	Kappa carrageenan-*g*-poly(acrylic acid) hydrogels	[38]
	**CO_2_**	In CO_2_ sensors, a pH sensitive hydrogel disc comes in contact with bicarbonate solution. On exposure to CO_2_, the pH of solution changes resulting in swelling or deswelling of the hydrogel which causes a change in pressure which is a measure of the partial pressure of CO_2_.	Poly(2-hydroxyethylmethacrylate)-*co*-(2-dimethylaminoethylmethacrylate)	[39]
	**Glucose**	Hydrogels show swelling in response to increased glucose concentration. The complex formed between glucose and phenylboronic acid drives the swelling of the hydrogels and consequently insulin release.	Poly(acrylamide)-*co*-(3-acrylamidophenylboronic acid)	[40]
	**Redox**	Disulfide linkages in reduction sensitive hydrogels cleave in the reductive environment (high level of glutathione concentration = 0.5–10 mM) in intracellular matrix and release bioactive molecules/drugs.	[poly(ethylene glycol) monomethyl ether]-graft-[disulfide linked poly(amido-amine)] and α-cyclodextrin	[41]
**Biological stimuli**	**Enzyme**	Enzymes cause hydrogel degradation and consequently the drug release. This is called a chemically controlled drug release mechanism.	Glycidylmethacrylate dextran-*g*-poly(acrylic acid)	[42]
	**Antigen**	Hydrogels sense the free antigen and undergo swelling followed by drug release.	*N*-succinimidylacrylate based antigen-antibody entrapment hydrogel	[13,18]
	**DNA**	Single stranded (ss) DNA grafted hydrogel probes show swelling in the presence of ssDNA.	Single stranded DNA probe-*g*-poly(acrylamide) hydrogels	[43]

**Table 2:** Composition of pH sensitive hydrogels along with the loaded drug and their functions.

Target	Compositions/Carrier		Drugs	Disease	Ref.
**Stomach**	**Chitosan**	Acrylic acid grafted chitosan/poly(vinyl pyrrolidone) cross-linked with glutaraldehyde and *N*,*N*-Methylene (bisacrylamide)	Clarithromycine	Peptic ulcer	[125]
		Chitosan cross-linked with citrate or tripolyphosphate	Metronidazole		[126]
		Chitosan/poly(vinyl pyrrolidone) blend cross-linked with glutaraldehyde	Amoxicilin		[127]
**Intestine**	Hemicellulose	Hemicellulose-*co*-acrylic acid	Theophylline	Respiratory tract diseases	[128]
	Guar gum	Acrylic acid grafted Guar gum blended with β-cyclodextrin and cross-linked with tetraethyl orthosilicate	Dexamethasone	Ulcerative colitis, arthritis.	[97]
	Cyclodextrin	β-cyclodextrin-*co*-methacrylic acid	Atorvastatin	Various hyperlipidemias	[129]
	Poly(ethylene glycol)	Styrene-butadiene-styrene incorporated into methacrylic acid-*co*-poly(ethylene glycol)	Pregabalin	Epilepsy, neuropathic pain, etc.	[130]
	Poly(vinyl pyrrolidone)	Lignosulfonate grafted poly(acrylic acid)-*co*-poly(vinyl pyrrolidone)	Amoxicilin	Bacterial infections	[131]
	Chitosan	Acrylic acid grafted chitosan	Insulin	Diabetes	[132]
**Colon**	Starch	Acrylic acid grafted starch	Rutin	Inflammatory bowel disease, allergy, etc.	[133]
	Guar gum	Guar gum succinate blended sodium alginate cross-linked with barium ions	Ibuprofen	Anti-inflammatory/anti-analgesic drug	[134]
	Alginate	Sodium alginate cross-linked with calcium chloride	Hydrocortisone	Allergy, arthritis, asthma.	[135]
	Gelatin	β-cyclodextrin grafted gelatin cross-linked with oxidized dextrin	5-Fluorouracil	Cancer	[136]
	Dextran	Glycidyl methacrylate dextran and poly(acrylic acid)	5-Aminosalicylic acid	Ulcerative colitis and Crohn’s disease	[42]
	Chitosan	Chitosan blended with poly(vinyl alcohol) cross-linked with tetraethyl orthosilicate	Dexamethasone	Ulcerative colitis and arthritis	[110]

**Table 3:** pH of different body parts/tissues in human body.

Fluids Tissue/Cellular Compartment	pH Ranges	References
Saliva in baccul cavity	6.7–7.3	[139]
Stomach	2.0	[35]
Duodenum	5.0–8.0	
Jejunum	6.0–7.0	[140]
Ileum	7.0	
Cecum	6.4	[123,138]
Colon	7.0–7.5	[35]
Rectum	7.0	[140]
Vagina	4.0–5.0	[141]
Chronic wounds	5.4–7.4	[35]
Extracellular matrix in cancerous tissue	6.5–7.2	
Lysosomes	4.5–5.0	
Golgi bodies	6.4	
Early endosome	6.0–6.5	
Late endosome	5.0–6.0	
Blood	7.35–7.45	
Stratum corneum	5.0–6.0	[142]
