# Erratum: pH Sensitive Hydrogels in Drug Delivery: Brief History, Properties, Swelling, and Release Mechanism, Material Selection and Applications. *Polymers* 2017, *9*, 137

**DOI:** 10.3390/polym9060225

**Published:** 2017-06-14

**Authors:** Muhammad Rizwan, Rosiyah Yahya, Aziz Hassan, Muhammad Yar, Ahmad Danial Azzahari, Vidhya Selvanathan, Faridah Sonsudin, Cheyma Naceur Abouloula

**Affiliations:** 1 Department of Chemistry, Universiti Malaya, 50603 Kuala Lumpur, Malaysia; rizi_chem1981@hotmail.com (M.R.); ahassan@um.edu.my (A.H.); o_danny@siswa.um.edu.my (A.D.A.); s_vidhya@siswa.um.edu.my (V.S.); 2 Interdisciplinary Research Center in Biomedical Materials, COMSATS Institute of Information Technology, 54000 Lahore, Pakistan; drmyar@ciitlahore.edu.pk; 3 Centre for Foundation Studies in Science, Universiti Malaya, 50603 Kuala Lumpur, Malaysia; sfaridah@um.edu.my; 4 Department of physics, Faculty of Science Semlalia Marrakesh, Cadi Ayyad University, 40000 Marrakesh, Morocco; cheyma.naceur@ced.uca.ac.ma

The authors wish to make a change to their published paper [1]. Figure 6 should be replaced with that shown below. The authors apologize for any inconvenience caused.

The change does not affect the scientific results. The manuscript will be updated and the original will remain online on the article webpage http://www.mdpi.com/2073-4360/9/4/137.

## Figures and Tables

**Figure 6:**
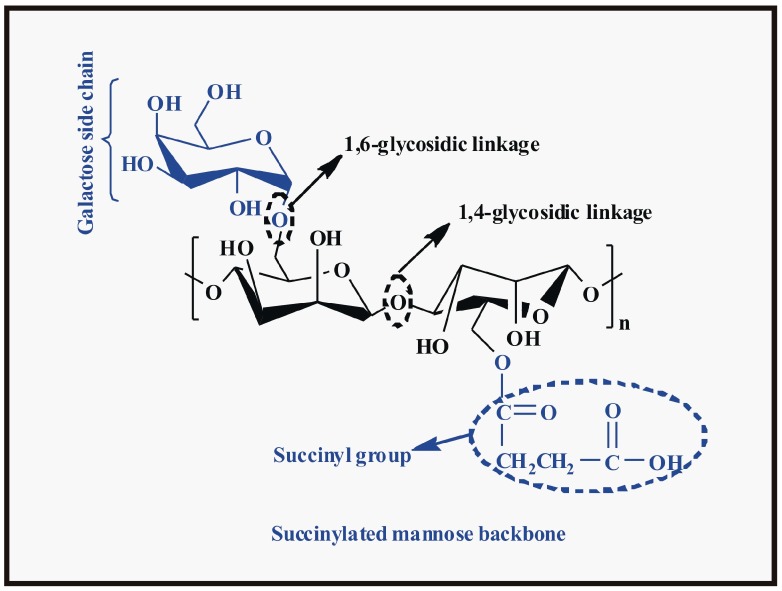
Structure of guar gum succinate.
